# Methyl Derivatives of Flavone as Potential Anti-Inflammatory Compounds

**DOI:** 10.3390/ijms26020729

**Published:** 2025-01-16

**Authors:** Dagmara Jaworska, Małgorzata Kłósek, Joanna Bronikowska, Agnieszka Krawczyk-Łebek, Martyna Perz, Edyta Kostrzewa-Susłow, Zenon P. Czuba

**Affiliations:** 1 Department of Microbiology and Immunology, Faculty of Medical Sciences in Zabrze, Medical University of Silesia in Katowice, Jordana 19, 41-808 Zabrze, Poland; mklosek@sum.edu.pl (M.K.); jbronikowska@sum.edu.pl (J.B.); zczuba@sum.edu.pl (Z.P.C.); 2 Department of Food Chemistry and Biocatalysis, Faculty of Biotechnology and Food Science, Wrocław University of Environmental and Life Sciences, 50-375 Wrocław, Poland; agnieszka.krawczyk-lebek@upwr.edu.pl (A.K.-Ł.); martyna.perz@umw.edu.pl (M.P.); edyta.kostrzewa-suslow@upwr.edu.pl (E.K.-S.); 3 Department of Biophysics and Neurobiology, Faculty of Medicine, Wrocław Medical University, Chałubińskiego 3A, 50-368 Wrocław, Poland

**Keywords:** flavone, methylflavones, anti-inflammation, cytokines, nitric oxide (NO)

## Abstract

Flavones are natural compounds that are broadly distributed in our diet. Their unique properties provide the possibility to control the immune system and the process of inflammation. A high intake of flavonoids, including flavones, may offer protection against reactive oxygen species, inflammation, and chronic diseases. In this research, we evaluated the anti-inflammatory effect of five methylflavones, 2′-methylflavone (**5C**), 3′-methylflavone (**6C**), 4′-methylflavone (**7C**), 6-methylflavone (**8C**), and 6-methyl-8-nitroflavone (**12C**), in lipopolysaccharide (LPS) stimulated RAW 264.7 cells (murine macrophage cell line). We estimated the nitrite concentration and detected reactive oxygen species using the chemiluminescence method. Moreover, we measured the production of pro-inflammatory cytokines using the Bio-Plex Magnetic Luminex Assay. As a result of our findings, we have established that some of the methyl derivatives of flavone inhibit nitric oxide (NO) production and chemiluminescence generated by LPS-stimulated macrophages, but they also have an influence on pro-inflammatory cytokines production. This study showed that 2′-methylflavone (**5C**) and 3′-methylflavone (**6C**) possess the strongest anti-inflammatory activity among all tested derivatives of flavone. In conclusion, our study demonstrated that methylflavones may be potentially valuable compounds for the alleviation of inflammatory reactions.

## 1. Introduction

Flavonoids are a group of polyphenols present in the human diet that are still a subject of increasing interest due to their wide biological properties. They can be found in almost all plant foods, and are especially abundant in the leaves, roots, flowers, and fruits [1,2,3]. This class of molecules is characterized by a C6–C3–C6 backbone structure, consisting of two aromatic rings (called A and B) linked to each other through three central carbon atoms that can form a heterocyclic ring (ring C) [1]. Flavonoids can be divided into several subclasses according to their structural differences, such as flavones, isoflavones, flavanones, flavonols, flavanols, etc. Moreover, substitutions and modifications in these rings can lead to further diversity among flavonoids, which determines different biological activity [4,5,6].

**Figure 1:**
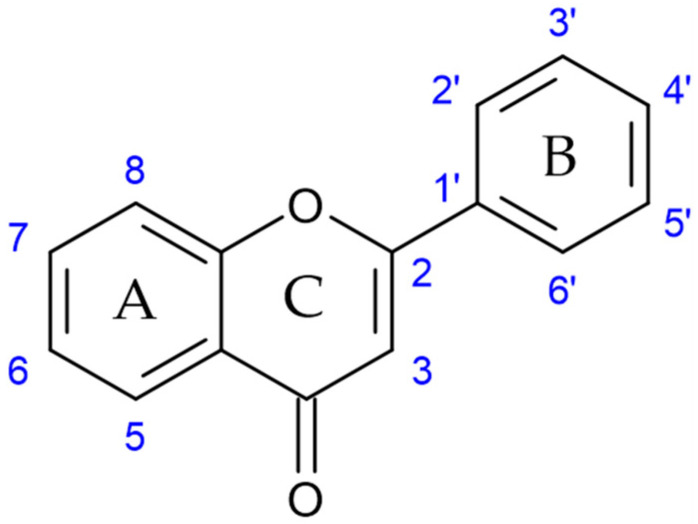
Structure of flavone.

Flavones are a subclass of flavonoids that are characterized by the presence of a double bond between C2 and C3 in the flavonoid skeleton and are oxidized at C4 (Figure 1) [7,8]. Flavones (from the Latin word flavus, meaning “yellow”) are colorless-to-yellow substances which occur mainly in flowers, leaves, and fruits. These compounds are distributed in the plant tissues in the form of glycosides and aglycones. They act as primary pigments or copigments in various parts of plants, acting as UVB protectants, natural pesticides, and even substances promoting the colonization of symbiotic bacteria or fungi [9]. This group of flavonoids occurs abundantly in *Ginkgo biloba*, celery, parsley, and chamomile [7]. Furthermore, they can be found in various types of tea and dry herbs [7,10]. Among fruits, flavones are most abundant in the citrus family. The most widely known flavone representatives comprise luteolin, apigenin, baicalein, genkwanin, and diosmetin [9,11].

Flavones represent one of the most studied subclasses of flavonoids due to their anti-inflammatory, antiallergic, antimutagenic, antithrombotic, antimicrobial, and anticarcinogenic activities [7,12,13,14,15,16]. They have been shown to have many benefits and, therefore, deserve further investigation.

Inflammation is a defense mechanism, typically induced by microbial infections, which involves the recruitment and activation of cells of innate and adaptive immunity. Microbial molecules that are recognized by the effector cells of innate immunity are called PAMPs (pathogen associated molecular patterns). Examples of such molecules are peptidoglycan, a component of the bacterial cell wall; LPS (lipopolysaccharide), found in the cell wall of Gram-negative bacteria; and teichoic acids, characteristic of Gram-positive bacteria [17,18].

There is no doubt that a controlled inflammatory reaction is beneficial for the organism; however, in some cases, it may produce a dysregulated response or be associated with a disruption of homeostasis. Consequently, the emergence of persistent inflammation leads to the pathogenesis of many types of disorders [19,20,21].

Chronic inflammation represents a reaction that is characterized by the continued recruitment of monocytes/macrophages and lymphocytes accompanied by tissue injury caused by sustained inflammatory response. Macrophages are key cellular elements of chronic inflammatory responses in tissues, which is associated with the amplified expression of proinflammatory mediators. Distinct populations of M1 and M2 macrophages regulate the chronic inflammatory environment (M1—classically activated phenotype, proinflammatory; M2—alternatively activated macrophages, anti-inflammatory). The macrophages play multiple roles in the inflammatory response and tissue repair, encouraging and discouraging these processes [22,23].

Many microbial products (like LPS) or cytokines such as interferon γ (IFN-γ) stimulate macrophages to increased production of nitric oxide (NO), reactive oxygen species (ROS), and an elevated production of proinflammatory cytokines such as interleukin-1 (IL-1), interleukin-6 (IL-6), interleukin-8 (IL-8), interleukin-12 (IL-12), and tumor necrosis factor-alpha (TNF-α) [24].

M1 macrophages undergo a metabolic shift during activation, involving the expression of inducible nitric oxide synthase (iNOS or NOS2), which generates large quantities of nitric oxide (NO). iNOS activity, unlike other nitric oxide synthases (NOS1—endothelial and NOS3—neuronal), strongly increases during stimulation with LPS. Constitutive nitric oxide synthases NOS1 and NOS3 usually generate NO at nanomolar concentrations. Under this physiological condition, NO regulates vasodilatation, neuronal transmission, and the inhibition of platelet aggregation. [25,26].

The role of NO in immunity is dual: It acts as both a toxic agent against infectious organisms and an immunoregulator. To perform these functions effectively, particularly as a toxic mediator, NO must be generated at micromolar levels for a sustained period of time [25,27].

During phagocytosis, macrophages and other phagocytic cells consume an increased amount of oxygen, which is related to the production of reactive oxygen species (ROS). This phenomenon associated with increased oxygen consumption is called “oxidative burst”. It can be studied in vitro, among others, through the chemiluminescence reaction. The ability of cells to undergo chemiluminescence is assessed after adding a stimulating agent (stimulated chemiluminescence). To enhance the reaction, two compounds with a high quantum yield can be used: luminol and lucigenin. These compounds easily transform into an excited state during the oxidation process, which allows the enhancement of the glowing effect [28]. The phenomenon of luminol-enhanced chemiluminescence was used to assess the oxygen burst in RAW 264.7 cells after stimulation with PMA (phorbol myristate acetate) in the presence of flavone or its methyl derivatives. Additionally, in a combination with flavone or its methyl derivatives, the nitric oxide production and proinflammatory cytokines expression were assessed.

The aim of this work was to evaluate the anti-inflammatory effect of five methylflavones, 2′-methylflavone (**5C**), 3′-methylflavone (**6C**), 4′-methylflavone (**7C**), 6-methylflavone (**8C**), and 6-methyl-8-nitroflavone (**12C**), on LPS-induced RAW 264.7 cells. The compounds used in this study have been previously characterized [29,30,31,32], but this study demonstrates their anti-inflammatory properties for the first time.

## 2. Results

### 2.1. The Cytotoxic Effect of 2′-Methylflavone (**5C**), 3′-Methylflavone (**6C**), 4′-Methylflavone (**7C**), 6-Methylflavone (**8C**), and 6-Methyl-8-nitroflavone (**12C**) on RAW 264.7 Macrophages

**Figure 2:**
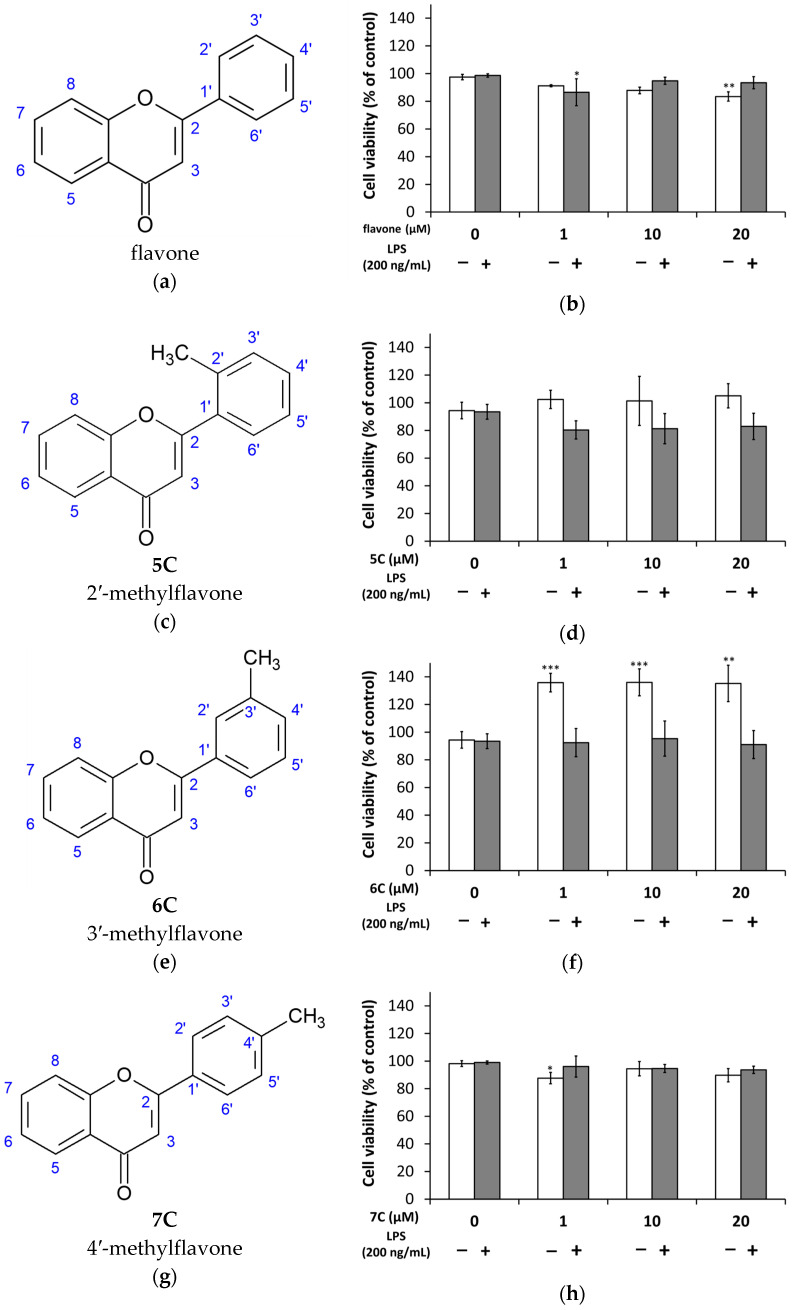

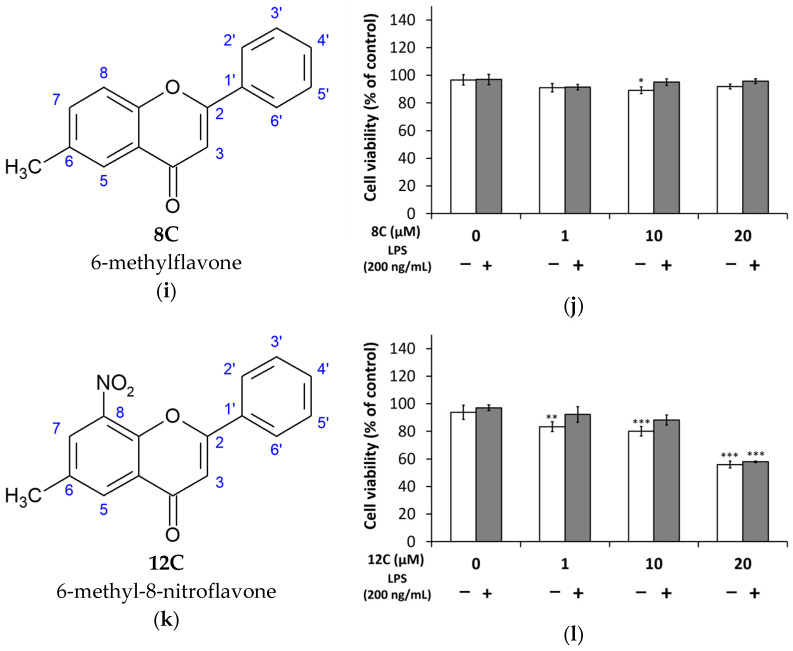
The viability of cell line RAW 264.7 in the presence of tested compounds. Chemical structure (**a**) and cytotoxic effect (**b**) of flavone, chemical structure (**c**) and cytotoxic effect (**d**) of 2′-methylflavone (**5C**), chemical structure (**e**) and cytotoxic effect (**f**) of 3′-methylflavone (**6C**), chemical structure (**g**) and cytotoxic effect (**h**) of 4′-methylflavone (**7C**), chemical structure (**i**) and cytotoxic effect (**j**) of 6-methylflavone (**8C**), chemical structure (**k**) and cytotoxic effect (**l**) of 6-methyl-8-nitroflavone (**12C**). The values represent means ± SD obtained from three independent experiments performed in quadruplicate (*n* = 12). The data were analyzed using analysis of variance (ANOVA) followed by Tukey’s test. * *p* < 0.05, ** *p* < 0.01, *** *p* < 0.001 compared to control cells.

The RAW 264.7 macrophages were incubated with 1 μM, 10 μM, and 20 μM of the listed compounds (with or without LPS): 2′-methylflavone (**5C**), 3′-methylflavone (**6C**), 4′-methylflavone (**7C**), 6-methylflavone (**8C**), and 6-methyl-8-nitroflavone (**12C**). The above concentration range was selected based on our previous experience with methyl derivatives of other groups of flavonoids, as well as a literature review in this area [14,33,34]. The results of the cytotoxicity assay (MTT) in relevance to the chemical structures of the tested compounds are presented (as a percentage of the control) in Figure 2.

As shown in Figure 2b, a statistically significant cytotoxic effect was observed for flavone at a concentration of 20 μM in cells not treated with LPS (83.42 ± 3.36%, *p* < 0.01) and at a concentration of 1 μM in cells treated with LPS (86.50 ± 9.73%, *p* < 0.05).

Interestingly, compound **6C** showed a proliferative effect on macrophages not treated with LPS (Figure 2f). As shown in Figure 2h, 1 μM of the **7C** compound induced a slight but significant cytotoxic effect in RAW 264.7 cells not treated with LPS (87.67 ± 4.10%, *p* < 0.05). However, the observed effects were the most severe in the cells treated with the **12C** compound (Figure 2l), for cells not treated with LPS (1 μM: 83.34 ± 3.58%, *p* < 0.01; 10 μM: 80.13 ± 3.38%, *p* < 0.001; 20 μM: 55.92 ± 2.38%, *p* < 0.001) and for cells treated with LPS (20 μM: 57.90 ± 0.48%, *p* < 0.001). The other compounds used did not show statistically significant cytotoxic effects on RAW 264.7 macrophages. The detailed data are available in the Supplementary Materials (Table S1).

### 2.2. The Nitric Oxide Production by LPS-Stimulated RAW 264.7 Cells in the Presence of Flavone and Its Methyl Derivatives: 2′-Methylflavone (**5C**), 3′-Methylflavone (**6C**), 4′-Methylflavone (**7C**), 6-Methylflavone (**8C**), and 6-Methyl-8-nitroflavone (**12C**)

**Figure 3:**
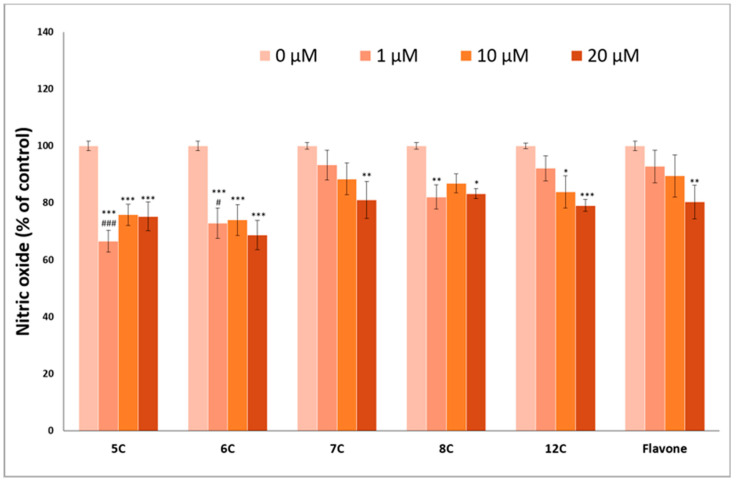
The production of nitric oxide by LPS-stimulated RAW 264.7 cells in the presence of flavone and its derivatives: 2′-methylflavone (**5C**), 3′-methylflavone (**6C**), 4′-methylflavone (**7C**), 6-methylflavone (**8C**), and 6-methyl-8-nitroflavone (**12C**). The values represent means ± SD obtained from three independent experiments performed in triplicate (*n* = 9). The data were analyzed using analysis of variance (ANOVA) followed by Tukey’s test. * *p* < 0.05, ** *p* < 0.01, *** *p* < 0.001 compared to LPS-stimulated cells. # *p* < 0.05, ### *p* < 0.001 compared to flavone and LPS -stimulated cells.

We evaluated the production of nitric oxide by LPS-stimulated RAW 264.7 cells in the presence of flavone and its derivatives: 2′-methylflavone (**5C**), 3′-methylflavone (**6C**), 4′-methylflavone (**7C**), 6-methylflavone (**8C**), and 6-methyl-8-nitroflavone (**12C**). Some of the tested compounds showed the ability to inhibit nitric oxide synthesis by activated RAW 264.7 cells. Among all tested flavone derivatives, **5C**, **6C**, and **8C** showed a significant inhibitory effect on NO production in the range of all tested concentrations. Flavone, as a precursor of obtained methyl derivatives, showed only a weak ability to inhibit nitric oxide synthesis. However, in comparison to flavone, only some concentrations of **5C** and **6C** derivatives exhibit a statistically significant inhibitory effect. This inhibitory effect of the tested compounds on NO production in RAW 264.7 is presented below as a percentage of the control (Figure 3). The detailed data are available in the Supplementary Materials (Table S2).

### 2.3. The Effect of Flavone and Its Methyl Derivatives, 2′-Methylflavone (**5C**), 3′-Methylflavone (**6C**), 4′-Methylflavone (**7C**), 6-Methylflavone (**8C**), and 6-Methyl-8-nitroflavone (**12C**), on Chemiluminescence of Activated RAW 264.7 Macrophages

In our study we evaluated the inhibitory effect on chemiluminescence of RAW 264.7 cells (preincubated with luminol and then stimulated with PMA, phorbol 12-myristate 13-acetate) in the presence of flavone and its derivatives: 2′-methylflavone (**5C**), 3′-methylflavone (**6C**), 4′-methylflavone (**7C**), 6-methylflavone (**8C**), and 6-methyl-8-nitroflavone (**12C**). The luminol-enhanced chemiluminescence reaction was used to assess oxygen burst in RAW 264.7 cells after stimulation with PMA in the presence of flavone or its methyl derivatives.

In the tests performed during our study, a significant inhibitory effect was observed compared to control cells for all tested compounds at concentrations above 10 µM.

Among all the methyl derivatives of flavone tested, only compounds **5C** and **6C** showed stronger activity than the original flavone itself.

**Figure 4:**
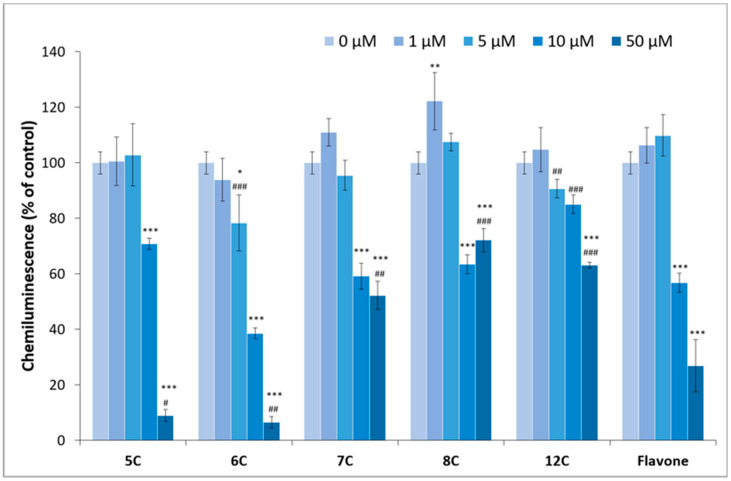
The chemiluminescence of RAW 264.7 cells in the presence of flavone and its derivatives: 2′-methylflavone (**5C**), 3′-methylflavone (**6C**), 4′-methylflavone (**7C**), 6-methylflavone (**8C**), and 6-methyl-8-nitroflavone (**12C**). The values represent means ± SD obtained from three independent experiments performed in triplicate (*n* = 9). The data were analyzed using analysis of variance (ANOVA) followed by Tukey’s test. * *p* < 0.05, ** *p* < 0.01, *** *p* < 0.001 compared to control cells. # *p* < 0.05, ## *p* < 0.01, ### *p* < 0.001 compared to flavone-stimulated cells.

The influence of the tested compounds on the chemiluminescence of RAW 264.7 macrophages is presented below as a percentage of the control (Figure 4). The detailed data are available in the Supplementary Materials (Table S3).

### 2.4. The Effect of Flavone and Its Methyl Derivatives, 2′-Methylflavone (**5C**), 3′-Methylflavone (**6C**), 4′-Methylflavone (**7C**), 6-Methylflavone (**8C**), and 6-Methyl-8-nitroflavone (**12C**), on the Release of IL-1α, IL-1β, IL-6, IL-12p70 and TNF-α by RAW 264.7 Macrophages

The results below are presented as percentages of the control due to differences in cytokine secretion by control cells depending on the plate which was examined. During the experiment, a very wide panel of methyl derivatives was analyzed by Bio-Plex Assay and only some of those derivatives are illustrated in the results. To compare the activity of different compounds on one graph, the values of secreted cytokines in pg/mL were compared to the appropriate controls, obtaining a percentage value (% of the control). The detailed data are available in the Supplementary Materials (Table S4).

**Figure 5:**
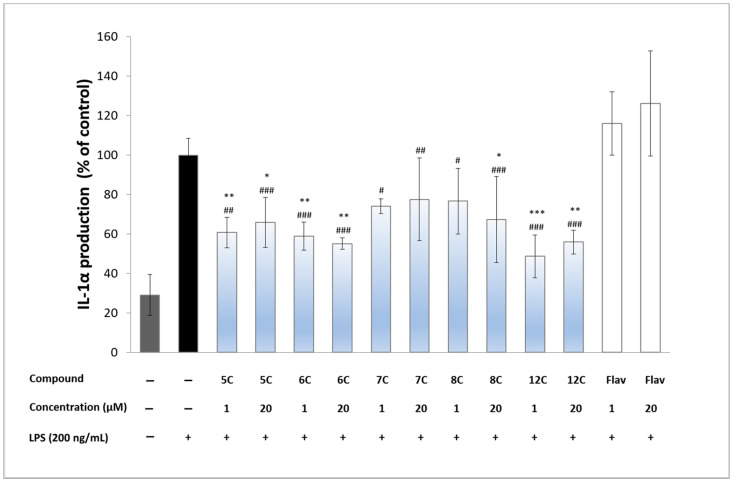
The IL-1α production by LPS stimulated RAW 264.7 cells in the presence of flavone and its derivatives: 2′-methylflavone (**5C**), 3′-methylflavone (**6C**), 4′-methylflavone (**7C**), 6-methylflavone (**8C**), and 6-methyl-8-nitroflavone (**12C**). The values represent means ± SD obtained from three independent experiments (*n* = 3). The data were analyzed using analysis of variance (ANOVA) followed by Tukey’s test. * *p* < 0.05, ** *p* < 0.01, *** *p* < 0.001 compared to LPS-stimulated cells. # *p* < 0.05, ## *p* < 0.01, ### *p* < 0.001 compared to flavone-stimulated cells.

The study results showed that compounds **5C**, **6C**, and **12C** significantly inhibited IL-1α concentration produced by RAW 264.7 cells compared to control cells stimulated with LPS, as well as compared to flavone. The strongest effect in this case was demonstrated by 3′-methylflavone (**6C**). The results obtained for 6-methyl-8-nitroflavone (**12C**) were inconclusive as increased inhibition was observed at a concentration of 1µM and reduced inhibition at a concentration of 20 µM. Such an effect may result from the cytotoxicity of this compound. Interestingly, flavone (the substrate) did not inhibit IL-1α concentration produced by RAW 264.7 cells; on the contrary, it even had a stimulating effect. The results showing an influence of the tested compounds on the IL-1α production by RAW 264.7 macrophages are presented below in Figure 5. The detailed data are available in the Supplementary Materials (Tables S4 and S5).

**Figure 6:**
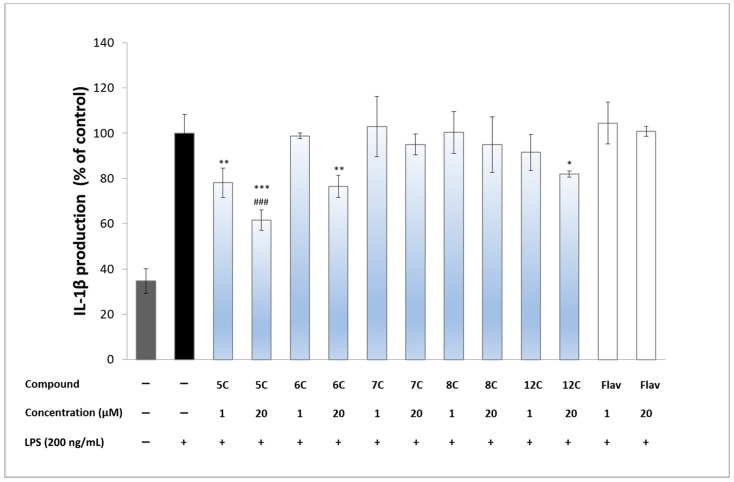
The IL-1β production by LPS stimulated RAW 264.7 cells in the presence of flavone and its derivatives: 2′-methylflavone (**5C**), 3′-methylflavone (**6C**), 4′-methylflavone (**7C**), 6-methylflavone (**8C**), and 6-methyl-8-nitroflavone (**12C**). The values represent means ± SD obtained from three independent experiments (*n* = 3). The data were analyzed using analysis of variance (ANOVA) followed by Tukey’s test. * *p* < 0.05, ** *p* < 0.01, *** *p* < 0.001 compared to LPS-stimulated cells. ### *p* < 0.001 compared to flavone stimulated cells.

Subsequently, the production of other members of the IL-1 family, including IL-1β, was also inhibited by some flavone methyl derivatives. In this case, the most active compound was 2′-methylflavone (**5C**), compared to LPS-stimulated control cells as well as compared to cells stimulated with flavone. 3′-methylflavone (**6C**) showed significant activity exclusively in the concentration of 20 µM. Compound **12C** (6-methyl-8-nitroflavone) showed only a slight significant effect compared to LPS-stimulated cells. The IL-1β production by LPS-stimulated RAW 264.7 macrophages in the presence of the tested compounds is presented below in Figure 6, as a percentage of the control. The detailed data are available in the Supplementary Materials (Tables S4 and S6).

In our study, we also evaluated the IL-6 production by RAW 264.7 macrophages. The results showed that compounds **5C** and **12C** significantly inhibited IL-6 concentration produced by RAW 264.7 cells compared to LPS-stimulated control cells and also compared to flavone. The strongest compound, in this case, was 6-methyl-8-nitroflavone (**12C**), but 2′-methylflavone (**5C**) also showed a significant inhibitory effect, compared to LPS-stimulated control cells as well as compared to flavone-stimulated cells. On the other hand, 3′-methylflavone (**6C**) and 4′-methylflavone (**7C**) showed small but significant activity only in the concentration of 20 µM, compared to LPS-stimulated control cells.

**Figure 7:**
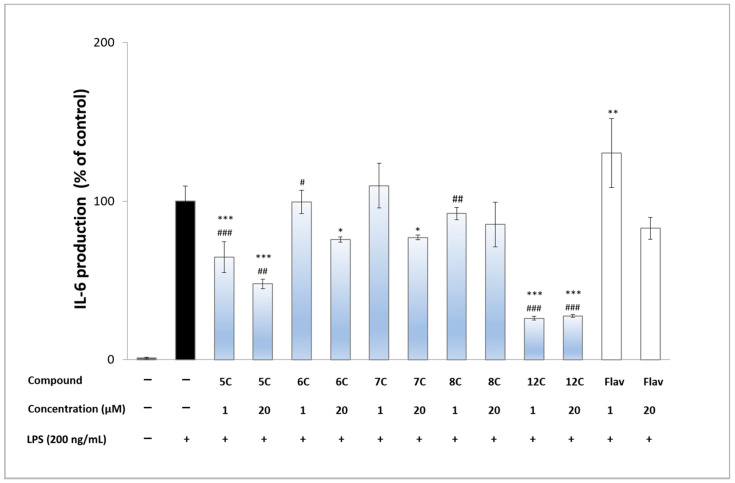
The IL-6 production by LPS stimulated RAW 264.7 cells in the presence of flavone and its derivatives: 2′-methylflavone (**5C**), 3′-methylflavone (**6C**), 4′-methylflavone (**7C**), 6-methylflavone (**8C**), and 6-methyl-8-nitroflavone (**12C**). The values represent means ± SD obtained from three independent experiments (*n* = 3). The data were analyzed using analysis of variance (ANOVA) followed by Tukey’s test. * *p* < 0.05, ** *p* < 0.01, *** *p* < 0.001 compared to LPS-stimulated cells. # *p* < 0.05, ## *p* < 0.01, ### *p* < 0.001 compared to flavone-stimulated cells.

The IL-6 production by LPS-stimulated RAW 264.7 macrophages in the presence of the tested compounds is presented below in Figure 7 as a percentage of the control. The detailed data are available in the Supplementary Materials (Tables S4 and S7).

Another example of a proinflammatory cytokine is IL-12, which is encoded by two genes: IL-12A (p35) and IL-12B (p40). There are two active forms of this cytokine—a heterodimer (p70) and a homodimer (p40). In this work, we assessed the production of IL-12p70 by LPS-stimulated RAW 264.7 macrophages. The results of the experiment showed that only 2′-methylflavone (**5C**) at a concentration of 20 μM turned out to be more active than the original flavone. However, compared to the control, 2′-methylflavone (**5C**), 6-methylflavone (**8C**), and 6-methyl-8-nitroflavone (**12C**) showed a significant inhibitory effect.

**Figure 8:**
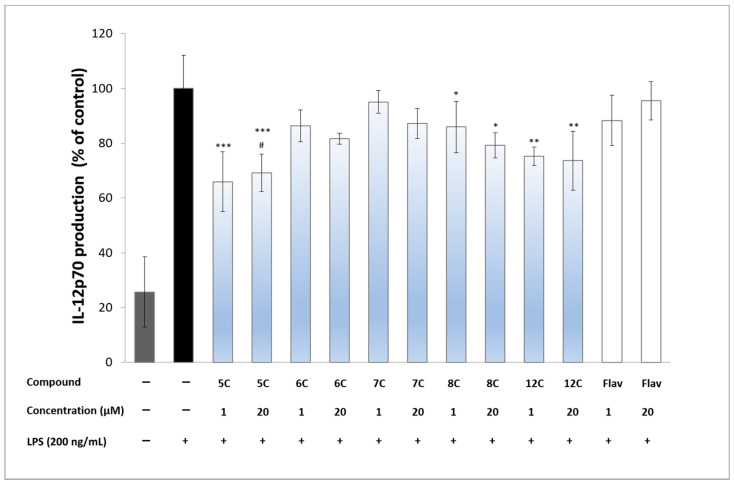
The IL-12p70 production by LPS stimulated RAW 264.7 cells in the presence of flavone and its derivatives: 2′-methylflavone (**5C**), 3′-methylflavone (**6C**), 4′-methylflavone (**7C**), 6-methylflavone (**8C**), and 6-methyl-8-nitroflavone (**12C**). The values represent means ± SD obtained from three independent experiments (*n* = 3). The data were analyzed using analysis of variance (ANOVA) followed by Tukey’s test. * *p* < 0.05, ** *p* < 0.01, *** *p* < 0.001 compared to LPS-stimulated cells. # *p* < 0.05, compared to flavone-stimulated cells.

The IL-12p70 production by LPS-stimulated RAW 264.7 macrophages in the presence of the tested compounds is presented below in Figure 8 as a percentage of the control. The detailed data are available in the Supplementary Materials (Tables S4 and S8).

One of the most important pro-inflammatory cytokines is TNF-α. The results of our studies indicate a significant impact of the tested compounds on the production of TNF-α by LPS-stimulated RAW 264.7 cells. All five tested methyl derivatives, as well as the flavone itself, had an inhibitory effect on the production of TNF. However, the most active compounds turned out to be 2′-methylflavone (**5C**) and 3′-methylflavone (**6C**), although their activity here is comparable to the original flavone.

**Figure 9:**
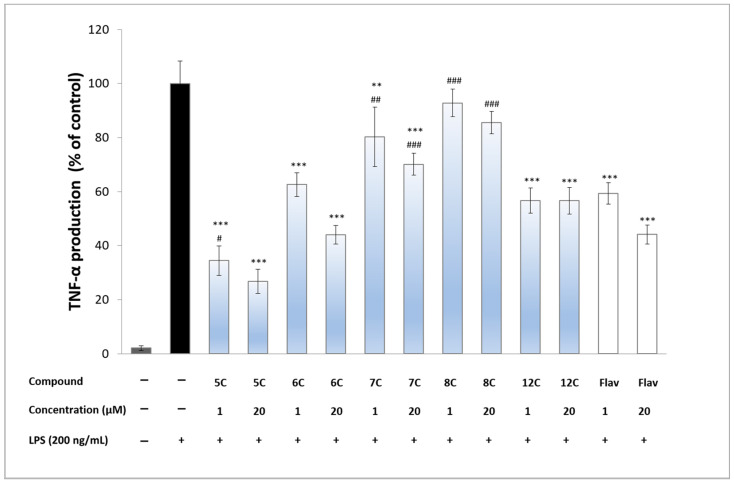
The TNF-α production by LPS stimulated RAW 264.7 cells in the presence of flavone and its derivatives: 2′-methylflavone (**5C**), 3′-methylflavone (**6C**), 4′-methylflavone (**7C**), 6-methylflavone (**8C**), and 6-methyl-8-nitroflavone (**12C**). The values represent means ± SD obtained from three independent experiments (*n* = 3). The data were analyzed using analysis of variance (ANOVA) followed by Tukey’s test. ** *p* < 0.01, *** *p* < 0.001 compared to LPS-stimulated cells. # *p* < 0.05, ## *p* < 0.01, ### *p* < 0.001 compared to flavone-stimulated cells.

The TNF-α production by LPS stimulated RAW 264.7 macrophages in the presence of the tested compounds is presented below in Figure 9 as a percentage of control. The detailed data are available in the Supplementary Materials (Tables S4 and S9).

## 3. Discussion

In recent years, there has been a growing interest in the ameliorating effects of dietary flavones in diseases associated with chronic inflammation. Our findings show the potential of the methyl derivatives of flavone as anti-inflammatory compounds which can be applied to the development of novel functional foods for the alleviation of inflammatory reactions.

Inflammation is a dynamic process triggered by the stimulation of both types of immunity, innate and acquired, in a response to pathogen invasion. The inflammatory process should, therefore, lead to the removal of the infectious agent and allow the tissue to return to its physiological state. Sometimes, however, a chronic, uncontrolled inflammatory reaction develops, which leads to extensive damage and pathological conditions accompanying disorders such as autoimmune diseases, metabolic diseases, and cancer [20,24,35,36]. Therefore, the inflammatory process, which should play a protective and regenerative role, can sometimes, paradoxically, lead to the exacerbation of the disease and even to death. During the development of the inflammatory response, when acute inflammation continues and turns into a chronic state, the composition of the white blood cells changes; therefore, macrophages and lymphocytes begin to replace short-lived neutrophils [37,38,39].

These cells produce a variety of inflammatory cytokines, chemokines, and oxidative enzymes that lead to the progression of tissue damage and secondary repair, including fibrosis and granuloma formation [23,33].

Macrophages exposed to immunogenic particles secrete pro-inflammatory cytokines, such as IL-1, IL-6, TNFα, and IL-8, IL-12, and chemokines, but also mediators such as nitric oxide (NO) and reactive oxygen species (ROS), leukotrienes, and prostaglandins. All of these molecules, cooperating with other cell products, induce vascular permeability and cell recruitment to the site of inflammation and also participate in the regulation of the immune response. However, their overproduction may lead to many disorders, for example, sepsis, chronic inflammation, and pain, as well as autoimmune diseases [22,23,24,40].

Flavonoids are a class of secondary plant metabolites present in different concentrations and forms in roots, leaves, flowers, and fruits. Among flavonoids, flavones represent one of the most studied groups, due to their antioxidant and anti-inflammatory properties, which make them an attractive target for the synthesis of more active derivatives and their further study [11,33].

It has already been established that some naturally occurring representatives of flavones demonstrate anti-inflammatory properties. One example of the anti-inflammatory effect of flavones is luteolin, which has been demonstrated to inhibit M1/M2 macrophage polarization, which, in turn, inhibits the inflammatory process [41]. Recent data showed the activity of luteolin-7-O-glucoside in the reduction of oxidative stress and inflammatory mechanisms in endothelial cells cultured in vitro. These anti-inflammatory properties can correlate with the cardiovascular benefits of this flavone [42]. Some sources also report that high luteolin intake can protect against and alleviate neurodegenerative diseases [43].

Moreover, another member of the flavones family, apigenin, has been shown to significantly suppress the inflammatory and allergic responses of RAW264.7 and RBL cells, thus potentially serving as a preventive and therapeutic agent for immune-related diseases [15]. In another study, apigenin effectively downregulated the expression and secretion of pro-inflammatory cytokines and was found effective against psoriasis in both in vitro and in vivo models [44].

Recently, it was established that the antioxidant activity of flavones could be related to the presence of a hydroxyl group situated on the B ring, much more than the A-ring substitution. Therefore, the type of the B-ring substitution is considered as a determinant of antiradical potency; just one hydroxyl group located there guarantees significant scavenging potential, especially in the position C4′. Generally, flavones belong to a group of flavonoids that show great capacity to scavenge free radicals and play a crucial role as a source of antioxidants. Their biological activity is related strictly to their molecular structure and depends on both the number and the place of attachment of functional groups [45].

Naturally occurring flavonoids exist in plants in either a glycosylated or methylated form, because these structures are more stable and also bioactive. Numerous studies have already shown that methylated flavonoids have significantly better biological properties than unmethylated forms. Methylation enhances the entry of flavonoids into cells and prevents their degradation [13,46]. Moreover, methylation increases the hydrophobicity of flavonoids and improves their affinity for the cell membrane. When tested in fibroblasts and astrocytes, the uptake of methylated flavanols and flavonols was higher than that of unmethylated ones [47]. However, there are no extensive studies on C-methylated flavonoids and the comparison of O-methylated versus C-methylated compounds. This situation is probably related to the rare occurrence of C-methylated flavonoids in nature; only a few plants contain large amounts of methylated compounds. For example, the genus *Citrus* is the most abundant natural source of methylated flavonoids, especially methylated flavones and flavonols. It contains both C-methylated and O-methylated flavones [46].

Studies focusing on the methylation of flavones have shown that this process leads to the formation of derivatives that are protected from extensive, rapid hepatic metabolism. Methylation also significantly increases their metabolic stability and enhances the membrane transport because of altered interaction with biological membranes [13,32,46,47,48,49].

Currently, there are not many studies on the biological activity of natural or synthetic methylflavones. Some reports concern the activity of methylflavones as a class of ligands acting on GABA(A) receptors and possessing anxiolytic, antidepressant, and anticonvulsant effects [49,50]. Clarkson et al. demonstrated that 2′-methoxy-6-methylflavone increases tonic GABA(A) receptor currents, and in the case of extrafocal ischemia, reduces infarct volume and improves recovery. Moreover, they investigated the anti-inflammatory effects of this compound, and in a macrophage cell line, they showed that 2′-methoxy-6-methylflavone could inhibit the LPS-induced increase in NF-κB (nuclear factor kappa-light-chain-enhancer of activated B cells) activity. Meanwhile, in stroke animals, this compound significantly decreased the circulating IL1-β, TNF-α and IFN-γ levels. Therefore, they hypothesized that this methylflavone, administered immediately after stroke, may increase neuroprotection [51].

Another group (Yue et al.) tested three structurally related 6-C-methyl flavones isolated from *Pinus densata* for their ability to inhibit proliferation and induce apoptosis in the HL-60 human leukemia cell line. They proved that some of these compounds exhibited significant anticancer activity via the mitochondrial caspase-3-dependent apoptosis pathway [52].

Synthetic 3-methylflavones with various substitutions on rings A and B were also evaluated for antioxidant activity and antibacterial action against Gram-positive and Gram-negative bacteria. It was discovered by Jayashree et al. that five of the tested compounds exhibited DPPH radical scavenging activity, and the same compounds also exhibited a 50–100% growth in the inhibition of Gram-positive but not Gram-negative bacteria [53].

In our work, we have estimated an anti-inflammatory effect of five methylflavones, 2′-methylflavone (**5C**), 3′-methylflavone (**6C**), 4′-methylflavone (**7C**), 6-methylflavone (**8C**), and 6-methyl-8-nitroflavone (**12C**), on LPS-induced RAW 264.7 cells. According to an extensive literature review, the biological activity of all the above-mentioned products have not been described in the literature yet. However, based on our research, some of these compounds can be considered as potentially bioactive.

For the first time, we have shown that some of the methyl derivatives of flavone inhibit nitric oxide production and the chemiluminescence of LPS-stimulated RAW 264.7 macrophages. This research demonstrated that 2′-methylflavone (**5C**) and 3′-methylflavone (**6C**) possess the strongest anti-inflammatory activity among all tested compounds.

Moreover, 2′-methylflavone (**5C**) and 3′-methylflavone (**6C**) inhibited most strongly the production of IL-1α, IL-1β, IL-6, and TNFα by the stimulated macrophages.

The anti-inflammatory effects of flavones and their derivatives can be attributed to various pathways, including the inactivation of transcription factors responsible for the induction of inflammation and the production of pro-inflammatory cytokines. The key signaling pathways contributing to inflammation impacted by flavones include NF-κB, MAPK (Mitogen activated protein kinase), STAT (signal transducer and activator of transcription), Nrf2 (Nuclear factor erythroid 2—related factor), and the inflammasome pathway [14,19,33].

The anti-inflammatory effects of flavones have been mainly related to the suppression of the NF-κB signal transduction pathway. It has been demonstrated that flavones can act as antagonists of NF-κB, which downregulates the production of iNOS and COX-2 [54]. It has been reported that apigenin (4′,5,7-trihydroxyflavone) inhibits the phosphorylation and degradation of IκB (IkappaB kinase), leading to the suppression of NF-κB activation [55]. Another interesting example of the anti-inflammatory effect of flavones is an action of diosmetin (5,7,3′-trihydroxy-4′-methoxyflavone), which has been shown to inhibit the translocation of NF-κB from the cytoplasm to the nucleus and increase the expression of the NF-κB inhibitor gene [56].

In relation to methylflavones, there are only a few publications describing their anti-inflammatory effects [50,51,53], but, in particular, these effects at the cellular level have not yet been elucidated. Future studies in this field will likely investigate which pathways are modulated by methyl derivatives of flavone. Leaving aside the results of similar studies, we can only hypothesize that these compounds, as other flavones, act in relation to the suppression of the NF-κB signal transduction pathway.

Moreover, one of the effects observed in our yet unpublished studies is an analogous proliferative effect, as well as a similar anti-inflammatory action in the corresponding methyl derivatives of flavone and chalcone. This is particularly clear in relation to the 3′-methylflavone (**6C**) derivative. Referring to this result, we may hypothesize that the mechanism of action of some methyl derivatives of flavone could be based on the electron delocalization across the molecule, breakdown of the C ring, and formation of an active chalcone with the same methyl substituent.

One of the tested compounds (6-methyl-8-nitroflavone (**12C**)) showed moderate anti-inflammatory activity; however, its action could be also an effect of the cytotoxicity demonstrated in the MTT test.

Different substitution at the flavones scaffold can induce diverse effects, which are often exerted through their DNA binding ability, because the structure facilitates intercalation between base pairs. Cardenas et al. have discovered the anti-proliferative action of several flavonoid derivatives in human and murine cell lines; this study indicated that a nitro group at position 4′ of the flavone yielded a very active anti-proliferative compound [57]. Perhaps flavone derivatives containing a nitro group could find an application in anticancer therapy due to their anti-proliferative activity. However, this requires further research and an assessment of their safety.

In this study, we evaluated the effect of different methyl derivatives of flavone on major mediators of the inflammatory reaction in RAW 264.7 cells. Although more extensive and additional research is needed to support this hypothesis, we can propose these compounds as potential candidates for alleviating immune-related diseases.

Moreover, the latest research in the field of flavonoids shows that many synthetic derivatives of flavone can demonstrate antiproliferative, anticancer, anti-inflammatory, antioxidant, and antimicrobial properties. Some of the recent papers regarding the modifications of the flavones skeleton are presented and cited below.

Daviaud et al. demonstrated the potential of polymethoxyflavones and polyacetylated flavones as analgesic and anti-inflammatory compounds in mice. This study suggested disruption in the production of inflammatory mediators such as COX-2, TNF-α, and IL-6 and as well as in the MAPK pathway [58]. Furthermore, in another study, new heterocyclic derivatives of flavone were synthesized and it was found out that the presence of phenoxy groups at selected positions of the A and B rings is crucial for cancer-selective cytotoxicity [59]. Khedera et al. synthesized new alkyl and ester derivatives based on the 3-hydroxy-4′-dimethylamino flavone. Some of these compounds showed high anti-fungal activity [60]. Another study showed that modifications introduced to the eupatilin structure improved the characteristics of this compound. The usefulness of the tested derivative (DA-6034, 7-carboxymethyloxy-3′,4′,5-trimethoxy flavone) was demonstrated, in particular, in chronic pancreatitis in a mouse model. The inhibition of NF-κB activation and reduction of the production of proinflammatory cytokines have been also demonstrated [61].

The publications cited above provide insight for optimization through pharmacomodulation and molecular docking to develop innovative, multi-target drugs. Such research constitutes an interesting solution for the development of new drugs in the nearest future. Moreover, the studies indicate that introducing new groups into already existing compounds of flavonoid origin in order to improve their properties may be an interesting and promising strategy.

## 4. Materials and Methods

### 4.1. Synthesis of Methyl Derivatives of Flavone

**Scheme 1:**
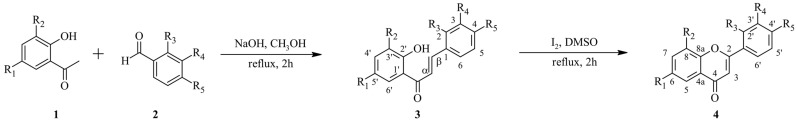
Synthesis of methyl derivatives of flavones.

The following compounds were used for the experiments: 2′-methylflavone (**5C**), 3′-methylflavone (**6C**), 4′-methylflavone (**7C**), 6-methylflavone (**8C**), and 6-methyl-8-nitroflavone (**12C**). They were obtained as a result of a two-step synthesis (Scheme 1), as described in our previous works [29,30,31,32].

In the first stage, Claisen–Schmidt condensation reactions were performed using several starting materials: 2′-hydroxyacetophenone with various methylbenzaldehydes (ortho, meta, para), 2′-hydroxy-5′-methylacetophenone with benzaldehyde, and 2′-hydroxy-5′-methyl-3′-nitroacetophenone with benzaldehyde. These reactions yielded the corresponding 2′-hydroxychalcones (2′-hydroxy-2-methylchalcone—**5A**, 2′-hydroxy-3-methylchalcone—**6A**, 2′-hydroxy-4-methylchalcone—**7A**, 2′-hydroxy-5′-methylchalcone—**8A**, and 2′-hydroxy-5′-methyl-3′-nitrochalcone—**12C**) containing methyl groups at different positions. Synthetic substrates purchased from Sigma-Aldrich (St. Louis, MO, USA) were dissolved in methanol, and the reactions were carried out in an alkaline medium (sodium hydroxide) for 2 h at elevated temperature by heating the mixture under reflux. After acidifying the reaction mixture with hydrochloric acid, the products were crystallized from 96% ethanol (**5A**—64% yield, **6A**—57% yield, **7A**—66% yield, **8A**—50% yield, and **12C**—75% yield). Then, flavones (**5C**, **6C**, **7C**, **8C**, and **12C**) were obtained from the appropriate chalcones (**5A**, **6A**, **7A**, **8A**, and **12A**) by oxidative cyclization, which lasted 2 h under reflux at 125 °C in an oil bath, in the presence of catalytic amounts of iodine, using dimethyl sulfoxide as a solvent for the reagents. Synthesis products were extracted from reaction mixture using methylene chloride and crystallized from 96% ethanol (**5C**—46% yield, **6C**—65% yield, **7C**—70% yield, **8C**—67% yield, and **12C**—90% yield).

All compounds are >98% pure by HPLC analysis. HPLC analyses were carried out using a Dionex Ultimate 3000 instrument (Thermo Fisher Scientific, Waltham, MA, USA) with a diode array detector using an analytical octadecylsilica (ODS) 2 column (4.6 × 250 mm, Waters, Milford, MA, USA) and pre-column. The gradient program was as follows: initial conditions—32.5% B in A, 4 min—40% B in A, 8 min—40% B in A, 10 min—45% B in A, 15 min—95% B in A, 18 min—95% B in A, 19 min—32.5% B in A, and 23 min—32.5% B in A. The flow rate was 1 mL/min, the injection volume was 5 µL, and the detection wavelength was 280 nm. NMR analyses were performed using a DRX Avance^TM^ 600 MHz NMR spectrometer (Bruker, Billerica, MA, USA). Molecular formulas of all products were confirmed by analysis performed on the LC-MS 8045 SHIMADZU Triple Quadrupole Liquid Chromatograph Mass Spectrometer (Shimazu Corporation, Kyoto, Japan) with electrospray ionization (ESI) source.

The data describing obtained synthesis products are shown below:

2′-Methylflavone (**5C**). ESI/MS *m*/*z* 237.1 ([M + H]^+^, C_16_H_12_O_2_); ^1^H NMR (600 MHz, acetone-d6) δ (ppm): 8.15 (1H, dd, *J* = 7.9, 1.7, H-5), 7.82 (1H, ddd, *J* = 8.7, 7.2, 1.7, H-7), 7.64 (2H, m, H-8 and H-6′), 7.50 (2H, m, H-6 and H-4′), 7.40 (2H, m, H-3′ and H-5′), 6.42 (1H, s, H-3), 2.51 (3H, s, C-2′-CH_3_); ^13^C NMR (151 MHz, acetone-d6) δ (ppm): 177.8 (C-4), 166.7 (C-2), 157.4 (C-8a), 134.9 (C-7), 137.7 (C-2′), 133.7 (C-1′), 132.1 (C-3′), 131.6 (C-4′), 130.2 (C-6′), 127.1 (C-5′), 126.2 (C-6), 126.0 (C-5), 124.7 (C-4a), 119.2 (C-8), 112.4 (C-3), 20.5 (C-2′-CH_3_).
3′-Methylflavone (**6C**). ESI/MS *m*/*z* 237.1 ([M + H]^+^, C_16_H_12_O_2_); ^1^H NMR (600 MHz, acetone-d6) δ (ppm): 8.12 (1H, dd, *J* = 7.9, 1.6, H-5), 7.92 (1H, s, H-2′), 7.88 (1H, d, *J* = 7.7, H-6′), 7.81 (1H, ddd, *J* = 8.6, 7.1, 1.7, H-7), 7.73 (1H, dd, *J* = 8.4, 0.9, H-8), 7.48 (2H, m, H-5′ and H-6), 7.42 (1H, d, *J* = 7.5, H-4′), 6.85 (1H, s, H-3), 2.45 (3H, s, C-3′-CH_3_); ^13^C NMR (151 MHz, acetone-d6) δ (ppm): 177.7 (C-4), 164.9 (C-2), 157.1 (C-8a), 139.8 (C-3′), 134.8 (C-7), 133.2 (C-4′), 132.7 (C-1′), 129.9 (C-5′), 127.7 (C-2′), 126.1 (C-6), 126.0 (C-5), 124.9 (C-4a), 124.4 (C-6′), 119.2 (C-8), 107.9 (C-3), 21.4 (C-2′-CH_3_).
4′-Methylflavone (**7C**). ESI/MS *m*/*z* 237.1 ([M + H]^+^, C_16_H_12_O_2_); ^1^H NMR (600 MHz, acetone-d6) δ (ppm): 8.12 (1H, dd, *J* = 7.9, 1.6, H-5), 7.99 (2H, d, *J* = 8.3, H-2′ and 6′), 7.81 (1H, ddd, *J* = 8.7, 7.1, 1.7, H-7), 7.73 (1H, d, *J* = 8.4, H-8), 7.48 (1H, m, H-6), 7.41 (2H, d, *J* = 8.0, H-3′ and H-5′), 6.82 (1H, s, H-3), 2.43 (3H, s, C-3′-CH_3_); ^13^C NMR (151 MHz, acetone-d6) δ (ppm): 177.9 (C-4), 164.1 (C-2), 157.2 (C-8a), 143.1 (C-4′), 134.7 (C-7), 130.6 (C-3′ and C-5′), 127.2 (C-2′ and C-6′), 126.1 (C-6), 126.0 (C-5), 124.9 (C-4a), 123.0 (C-1′), 119.2 (C-8), 107.4 (C-3), 21.4 (C-2′-CH_3_).
6-Methylflavone (**8C**). ESI/MS *m*/*z* 237.1 ([M + H]^+^, C_16_H_12_O_2_); ^1^H NMR (600 MHz, acetone-d6) δ (ppm): 8.12 (2H, m, H-2′ and H-6′), 7.94 (1H, s, H-5), 7.67 (2H, s, H-7 and H-8); 7.63 (3H, m, H-3′, H-4′, H-5′); 6.88 (1H, s, H-3); 2.50 (3H, s, C6-CH_3_); ^13^C NMR (151 MHz, acetone-d6) δ (ppm):178.0 (C-4), 163.8 (C-2), 155.4 (C-8a), 136.1 (C-6), 135.9 (C-8), 132.8 (C-1′), 132.4 (C-4′), 130.0 (C-3′ and C-5′), 127.2 (C-2′ and C-6′), 125.3 (C-5), 124.5 (C-4a), 119.0 (C-7), 107.8 (C-3), 20.9 (C6-CH_3_).
6-Methyl-8-nitroflavone (**12C**). ESI/MS *m*/*z* 282.1 ([M + H]^+^, C_16_H_11_NO_4_; ^1^H NMR (600 MHz; acetone-d6) δ (ppm): 8.35 (1H, dd, *J* = 2.2, 0.5 Hz, H-7), 8.24 (1H, dd, *J* = 2.2, 0.8 Hz, H-5), 8.15 (2H, ddd, *J* = 5.7, 4.3, 2.5 Hz, H-2′, H-6′), 7.65–7.61 (3H, m, H-3′, H-4′, H-5′), 7.01 (1H, s, H-3), 2.59 (3H, s, C-6-CH_3_); ^13^C NMR (151 MHz, acetone-d6) δ (ppm): 176.23 (C-4), 163.90 (C-2), 147.56 (C-8a), 139.61 (C-8), 136.24 (C-6), 133.00 (C-4′), 131.92 (C-1′), 131.57 (C-5), 131.53 (C-7), 130.11, (C-3′, C-5′), 127.45 (C-2′, C-6′), 126.31 (C-4a), 107.97 (C-3), 20.60 (C-6-CH_3_).

The structures of the tested flavones are shown below in Table 1.

### 4.2. Cell Culture

In the experiments, the RAW 264.7 mouse macrophages cell line was used (ATCC, American Type Culture Collection, Manassas, VA, USA). Cell culture was carried out in polystyrene bottles (Nunc A/S Roskilde, Denmark) in DMEM culture medium with the addition of L-glutamine (2 mM), penicillin (100 IU/mL), streptomycin (100 μg/mL), and 10% fetal calf serum (FBS). Cells were cultured continuously at 37 °C, in the atmosphere of 5% CO_2_, in an incubator at 100% relative humidity, and passage was performed 2–3 times a week. The attached cells were mechanically detached from the bottom of the vessel, and then, suspensions were prepared. The density of suspensions was assessed using the microscopic method using a Bürker chamber (Merck KGaA, Darmstadt, Germany). A suspension with a density of 1 × 10^6^/mL was used for further experiments.

During the experiments, the cells were activated with LPS (lipopolysaccharide) at a concentration of 200 ng/mL isolated from *Escherichia coli* of serotype O111:B4.

### 4.3. Cell Viability Assay

The cytotoxic effect of the tested compounds on RAW 264.7 cells was determined using the bromo-3-(4,5-dimethylthiazol-2-yl)-2,5-diphenyltetrazolium (MTT) assay obtained from Sigma Aldrich (St. Louis, MO, USA).

The MTT test is based on the measurement of mitochondrial dehydrogenase activity. After 24 h stimulation with methyl derivatives 2′-methylflavone (**5C**), 3′-methylflavone (**6C**), 4′-methylflavone (**7C**), 6-methylflavone (**8C**), and 6-methyl-8-nitroflavone (**12C**) at concentrations of 1 μM, 10 μM, and 20 μM, the cells were washed and the MTT solution was added to them, obtaining a final concentration of 1.1 mM, and then, the culture was continued for another 4 h. After this time, the cells were centrifuged, the supernatant was collected, and dimethyl sulfoxide (DMSO) was added to the adhered cells to extract the MTT formazan. The solution was taken after 20 min and the absorbance of the supernatant determined at a wavelength of 550 nm using an EonTM microplate spectrophotometer (BioTek Intruments, Winooski, VT, USA).

Based on the results obtained, the percentage of live cells was calculated in relation to the appropriate control, according to the following formula: cells viability (% of control) = (absorbance of tested wells × 100%)/absorbance of control wells.

### 4.4. Nitric Oxide Release Assay

Nitric oxide produced by the cells was determined by measuring the concentration of nitrite anion using Griess’ reagent. During the reaction, nitrite reacts with sulphanilic acid amide in an acidic environment. A diazo compound is then formed, which is coupled with N-(naphthyl)-ethylenediamine, and it gives a colored reaction product. A total 100 μL of supernatant from cell cultures stimulated with LPS and the tested compounds were used for the experiments. After mixing in a 1:1 ratio with Griess reagent in 96-well plates, the samples were incubated for 10 min at room temperature, and then, the absorbance was measured at a wavelength of 530 nm using an EonTM microplate spectrophotometer (BioTek Intruments, Winooski, VT, USA). The concentration of nitrite anions was determined on the basis of a standard curve prepared for sodium nitrite.

### 4.5. Chemiluminescence Detection

Chemiluminescence of RAW 264.7 cells was performed after a 5 min preincubation with luminol, and then, PMA solution (phorbol myristate acetate, Sigma Aldrich, St. Louis, MO, USA) was added, which induced chemiluminescence by activating protein kinase C. The determination was performed for four different concentrations of flavone and its methyl derivatives.

During the experiment, RAW 264.7 cells, as a suspension with a density of 1 × 10^7^/mL were placed in a 96-well plate, and then, tested compounds were added at appropriate concentrations. Final suspension of cells was 10^6^/mL (2 × 10^5^/well). Chemiluminescence was measured for 5 min with luminol at a final concentration of 10^−4^ mol/L. In the next step, the cells were stimulated with PMA with a final concentration of 8 × 10^−7^ mol/L and chemiluminescence was determined for another 30 min. The measurements were conducted using an LB 960 CentroXS3 luminometer (Berthold Technologies GmbH, Wildbad, Germany).

### 4.6. Bio-Plex Multiplex Immunoassay

The production of IL-1α, IL-1β, IL-6, IL-12p70, and TNF-α released from RAW 264.7 cells was determined after 24 h of stimulation with 2′-methylflavone (**5C**), 3′-methylflavone (**6C**), 4′-methylflavone (**7C**), 6-methylflavone (**8C**), 6-methyl-8-nitroflavone (**12C**), and flavone with or without LPS in the supernatants of cell culture. The measurements were conducted with the Bio-Plex Magnetic Luminex Assay (Bio-Rad Inc., Hercules, CA, USA) and the Bio-PlexTM 200 System (Bio-Rad Laboratories, Inc., Hercules, CA, USA).

Bio-Plex assays are bead-based experiments and can be performed in a mixed array (multiplexed). The reader combines two lasers, fluidics, and real-time digital signal processing to distinguish up to 100 different sets of color-coded polystyrene beads, each bearing a different antibody. Distinctly colored bead sets are created by the use of two fluorescent dyes at various ratios. To identify the different assays based on bead color and to quantify the analyte by measurement of the reporter dye, a dual detection based on flow cytometry technique is used.

In our experiments, sets of magnetic beads coated with antibodies specific for the analytes (specific cytokines) were added to the supernatants of cell cultures. Analytes (bead-linked) were detected using of a cocktail of biotinylated antibodies, which reacts with the streptavidin phycoerythrin conjugate. After each incubation, a magnetic washer (ELx 50, BioTek, Winooski, VT, USA) was used to remove additional beads.

In the Bio-Plex assays, serially diluted standards were used, to generate a calibration and standard curve. A regression analysis was then performed to predict the concentrations of the analyzed sample.

### 4.7. Statistical Analysis

All data are expressed as mean ± standard deviation (SD). In the case of cytotoxicity determination, the values represent means ± SD obtained from three independent experiments performed in quadruplicate (*n* = 12). Statistical significance from the control group was evaluated using two-way analysis of variance (ANOVA), followed by Tukey’s test, and *p*-values < 0.05 were considered significant. The homogeneity of variance was tested with Levene’s test.

In the chemiluminescence and in the nitric oxide assay, the values represent means ± SD obtained from three independent experiments performed in triplicate (*n* = 9). In the Bio-Plex Immunoassay, the values represent means ± SD obtained from three independent experiments (*n* = 3). Statistical significance from the control group and from the flavone was evaluated using one-way analysis of variance (ANOVA), followed by Tukey’s test, and *p*-values < 0.05 were considered significant. The homogeneity of variance was tested with Levene’s test.

The statistical analyses were performed using STATISTICA 13.3 software (StatSoft Inc., Tulsa, OK, USA).

## 5. Conclusions

Nowadays, the discovery of more specific immunomodulatory agents of plant origin that possess anti-inflammatory effect has attracted great attention. Among them, flavones constitute a group whose anti-inflammatory effect is indisputable.

In our study, we assessed anti-inflammatory effect of five methylflavones, 2′-methylflavone (**5C**), 3′-methylflavone (**6C**), 4′-methylflavone (**7C**), 6-methylflavone (**8C**), and 6-methyl-8-nitroflavone (**12C**), on LPS induced RAW 264.7 cells. Based on our results, we can conclude that 2′-methylflavone (**5C**) and 3′-methylflavone (**6C**) showed the strongest anti-inflammatory effects, suggesting their potential to mitigate inflammation-related health conditions and contribute to the prevention or management of chronic inflammatory diseases in the future.

In our future studies, we plan to extend our research beyond RAW 264.7 cells, to include other macrophages cell lines, as well as in vivo studies.

## Figures and Tables

**Table 1:** Structures of flavones: 2′-methylflavone (**5C**), 3′-methylflavone (**6C**), 4′-methylflavone (**7C**), 6-methylflavone (**8C**), and 6-methyl-8-nitroflavone (**12C**).

Compound	Structure	R_1_	R_2_	R_3_	R_4_	R_5_
**5C**	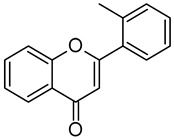	H	H	CH_3_	H	H
**6C**	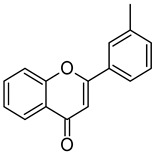	H	H	H	CH_3_	H
**7C**	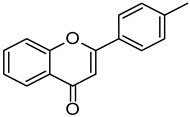	H	H	H	H	CH_3_
**8C**	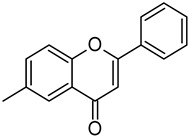	CH_3_	H	H	H	H
**12C**	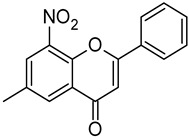	CH_3_	NO_2_	H	H	H

## Data Availability

All data are available from the corresponding author on special request.

## Supplementary Materials

The following supporting information can be downloaded at: https://www.mdpi.com/article/10.3390/ijms26020729/s1.

## References

[1] Wen K., Fang X., Yang J., Yao Y., Nandakumar K.S., Salem M.L., Cheng K. (2021). Recent Research on Flavonoids and Their Biomedical Applications. Curr. Med. Chem..

[2] Do Socorro Chagas M.S., Behrens M.D., Moragas-Tellis C.J., Penedo G.X.M., Silva A.R., Gonçalves-De-Albuquerque C.F. (2022). Flavonols and Flavones as Potential Anti-Inflammatory, Antioxidant, and Antibacterial Compounds. Oxidative Med. Cell. Longev..

[3] Shen N., Wang T., Gan Q., Liu S., Wang L., Jin B. (2022). Plant Flavonoids: Classification, Distribution, Biosynthesis, and Antioxidant Activity. Food Chem..

[4] Wang S., Alseekh S., Fernie A.R., Luo J. (2019). The Structure and Function of Major Plant Metabolite Modifications. Mol. Plant..

[5] Xie Y., Yang W., Tang F., Chen X., Ren L. (2015). Antibacterial Activities of Flavonoids: Structure-Activity Relationship and Mechanism. Curr. Med. Chem..

[6] Majee C., Mazumder R., Choudhary A.N. (2023). Salahuddin An Insight into the Hepatoprotective Activity and Structure-Activity Relationships of Flavonoids. Mini-Rev. Med. Chem..

[7] Hostetler G.L., Ralston R.A., Schwartz S.J. (2017). Flavones: Food Sources, Bioavailability, Metabolism, and Bioactivity. Adv. Nutr..

[8] Singh M., Kaur M., Silakari O. (2014). Flavones: An Important Scaffold for Medicinal Chemistry. Eur. J. Med. Chem..

[9] Panche A.N., Diwan A.D., Chandra S.R. (2016). Flavonoids: An Overview. J. Nutr. Sci..

[10] Diamond B.J., Bailey M.R. (2013). Ginkgo Biloba. Indications, Mechanisms, and Safety. Psychiatr. Clin. N. Am..

[11] Leonte D., Ungureanu D., Zaharia V. (2023). Flavones and Related Compounds: Synthesis and Biological Activity. Molecules.

[12] Hostetler G., Riedl K., Cardenas H., Diosa-Toro M., Arango D., Schwartz S., Doseff A.I. (2012). Flavone Deglycosylation Increases Their Anti-Inflammatory Activity and Absorption. Mol. Nutr. Food. Res..

[13] Walle T. (2007). Methylation of Dietary Flavones Greatly Improves Their Hepatic Metabolic Stability and Intestinal Absorption. Mol. Pharm..

[14] Kariagina A., Doseff A.I. (2022). Anti-Inflammatory Mechanisms of Dietary Flavones: Tapping into Nature to Control Chronic Inflammation in Obesity and Cancer. Int. J. Mol. Sci..

[15] Park C.H., Min S.Y., Yu H.W., Kim K., Kim S., Lee H.J., Kim J.H., Park Y.J. (2020). Effects of Apigenin on Rbl-2h3, Raw264.7, and Hacat Cells: Anti-Allergic, Anti-Inflammatory, and Skin-Protective Activities. Int. J. Mol. Sci..

[16] Serafini M., Peluso I., Raguzzini A. (2010). Flavonoids as Anti-Inflammatory Agents. Proc. Nutr. Soc..

[17] Coyne C.B., Zeh H.J., Lotze M.T., Tang D., Kang R. (2012). PAMPs and DAMPs: Signal 0s That Spur Autophagy and Immunity. Immunol. Rev..

[18] Roh J.S., Sohn D.H. (2018). Origin and List of DAMPS. Immune Netw..

[19] Zhang L., Xu L.Y., Tang F., Liu D., Zhao X.L., Zhang J.N., Xia J., Wu J.J., Yang Y., Peng C. (2024). New Perspectives on the Therapeutic Potential of Quercetin in Non-Communicable Diseases: Targeting Nrf2 to Counteract Oxidative Stress and Inflammation. J. Pharm. Anal..

[20] Kaur B., Singh P. (2022). Inflammation: Biochemistry, Cellular Targets, Anti-Inflammatory Agents and Challenges with Special Emphasis on Cyclooxygenase-2. Bioorg. Chem..

[21] Maleki S.J., Crespo J.F., Cabanillas B. (2019). Anti-Inflammatory Effects of Flavonoids. Food. Chem..

[22] Guha Ray A., Odum O.P., Wiseman D., Weinstock A. (2023). The Diverse Roles of Macrophages in Metabolic Inflammation and Its Resolution. Front. Cell Dev. Biol..

[23] Parisi L., Gini E., Baci D., Tremolati M., Fanuli M., Bassani B., Farronato G., Bruno A., Mortara L. (2018). Macrophage Polarization in Chronic Inflammatory Diseases: Killers or Builders?. J. Immunol. Res..

[24] Duque G.A., Descoteaux A. (2014). Macrophage Cytokines: Involvement in Immunity and Infectious Diseases. Front. Immunol..

[25] Fang F.C., Vazquez-Torres A. (2002). Nitric Oxide Production by Human Macrophages: There’s NO Doubt about It. Am. J. Physiol.-Lung Cell. Mol. Physiol..

[26] Palmieri E.M., McGinity C., Wink D.A., McVicar D.W. (2020). Nitric Oxide in Macrophage Immunometabolism: Hiding in Plain Sight. Metabolites.

[27] Levine A.B., Punihaole D., Levine T.B. (2012). Characterization of the Role of Nitric Oxide and Its Clinical Applications. Cardiology.

[28] Giussani A., Farahani P., Martínez-Muñoz D., Lundberg M., Lindh R., Roca-Sanjuán D. (2019). Molecular Basis of the Chemiluminescence Mechanism of Luminol. Chemistry.

[29] Krawczyk-łebek A., Dymarska M., Janeczko T., Kostrzewa-Susłow E. (2021). Fungal Biotransformation of 2′-Methylflavanone and 2′-Methylflavone as a Method to Obtain Glycosylated Derivatives. Int. J. Mol. Sci..

[30] Perz M., Krawczyk-Łebek A., Dymarska M., Janeczko T., Kostrzewa-Susłow E. (2023). Biotransformation of Flavonoids with -NO2, -CH3 Groups and -Br, -Cl Atoms by Entomopathogenic Filamentous Fungi. Int. J. Mol. Sci..

[31] Dymarska M., Grzeszczuk J., Urbaniak M., Janeczko T., Pląskowska E., Stępień Ł., Kostrzewa-Susłow E. (2017). Glycosylation of 6-Methylflavone by the Strain Isaria Fumosorosea KCH J2. PLoS ONE.

[32] Włoch A., Strugała-Danak P., Pruchnik H., Krawczyk-Łebek A., Szczecka K., Janeczko T., Kostrzewa-Susłow E. (2021). Interaction of 4′-Methylflavonoids with Biological Membranes, Liposomes, and Human Albumin. Sci. Rep..

[33] Wang X., Cao Y., Chen S., Lin J., Bian J., Huang D. (2021). Anti-Inflammation Activity of Flavones and Their Structure-Activity Relationship. J. Agric. Food Chem..

[34] Kłósek M., Krawczyk-Łebek A., Kostrzewa-Susłow E., Szliszka E., Bronikowska J., Jaworska D., Pietsz G., Czuba Z.P. (2023). In Vitro Anti-Inflammatory Activity of Methyl Derivatives of Flavanone. Molecules.

[35] Cobo G., Lindholm B., Stenvinkel P. (2018). Chronic Inflammation in End-Stage Renal Disease and Dialysis. Nephrol. Dial. Transpl..

[36] Tanaka T., Narazaki M., Masuda K., Kishimoto T. (2016). Regulation of IL-6 in Immunity and Diseases. Adv. Exp. Med. Biol..

[37] Germolec D.R., Shipkowski K.A., Frawley R.P., Evans E. (2018). Markers of Inflammation. Methods. Mol. Biol..

[38] Suzuki K. (2019). Chronic Inflammation as an Immunological Abnormality and Effectiveness of Exercise. Biomolecules.

[39] Arulselvan P., Fard M.T., Tan W.S., Gothai S., Fakurazi S., Norhaizan M.E., Kumar S.S. (2016). Role of Antioxidants and Natural Products in Inflammation. Oxidative Med. Cell. Longev..

[40] Doganyigit Z., Eroglu E., Akyuz E. (2022). Inflammatory Mediators of Cytokines and Chemokines in Sepsis: From Bench to Bedside. Hum. Exp. Toxicol..

[41] Wang S., Cao M., Xu S., Shi J., Mao X., Yao X., Liu C. (2020). Luteolin Alters Macrophage Polarization to Inhibit Inflammation. Inflammation.

[42] De Stefano A., Caporali S., Di Daniele N., Rovella V., Cardillo C., Schinzari F., Minieri M., Pieri M., Candi E., Bernardini S. (2021). Anti-Inflammatory and Proliferative Properties of Luteolin-7-O-Glucoside. Int. J. Mol. Sci..

[43] Daily J.W., Kang S., Park S. (2021). Protection against Alzheimer’s Disease by Luteolin: Role of Brain Glucose Regulation, Anti-Inflammatory Activity, and the Gut Microbiota-Liver-Brain Axis. BioFactors.

[44] Singh V.K., Sahoo D., Agrahari K., Khan A., Mukhopadhyay P., Chanda D., Yadav N.P. (2023). Anti-Inflammatory, Anti-Proliferative and Anti-Psoriatic Potential of Apigenin in RAW 264.7 Cells, HaCaT Cells and Psoriasis like Dermatitis in BALB/c Mice. Life Sci..

[45] Spiegel M., Andruniów T., Sroka Z. (2020). Flavones’ and Flavonols’ Antiradical Structure—Activity Relationship—A Quantum Chemical Study. Antioxidants.

[46] Wen L., Jiang Y., Yang J., Zhao Y., Tian M., Yang B. (2017). Structure, Bioactivity, and Synthesis of Methylated Flavonoids. Ann. N. Y. Acad. Sci..

[47] Spencer J.P.E., Abd El Mohsen M.M., Rice-Evans C. (2004). Cellular Uptake and Metabolism of Flavonoids and Their Metabolites: Implications for Their Bioactivity. Arch. Biochem. Biophys..

[48] Elkin Y.N., Kulesh N.I., Stepanova A.Y., Solovieva A.I., Kargin V.M., Manyakhin A.Y. (2018). Methylated Flavones of the Hairy Root Culture Scutellaria Baicalensis. J. Plant Physiol..

[49] Karim N., Gavande N., Wellendorph P., Johnston G.A.R., Hanrahan J.R., Chebib M. (2011). 3-Hydroxy-2′-Methoxy-6-Methylflavone: A Potent Anxiolytic with a Unique Selectivity Profile at GABA A Receptor Subtypes. Biochem. Pharmacol..

[50] Karim N., Khan I., Ahmad N., Umar M.N., Gavande N. (2017). Antidepressant, Anticonvulsant and Antinociceptive Effects of 3′-Methoxy-6-Methylflavone and 3′-Hydroxy-6-Methylflavone May Involve GABAergic Mechanisms. Pharmacol. Rep..

[51] Clarkson A.N., Boothman-Burrell L., Dósa Z., Nagaraja R.Y., Jin L., Parker K., van Nieuwenhuijzen P.S., Neumann S., Gowing E.K., Gavande N. (2019). The Flavonoid, 2′-Methoxy-6-Methylflavone, Affords Neuroprotection Following Focal Cerebral Ischaemia. J. Cereb. Blood Flow Metab..

[52] Yue R., Li B., Shen Y., Zeng H., Yuan H., He Y., Zhang W. (2013). 6-C-Methyl Flavonoids Isolated from Pinus Densata Inhibit the Proliferation and Promote the Apoptosis of the HL-60 Human Promyelocytic Leukaemia Cell Line. Planta Medica.

[53] Jayashree B.S., Alam A., Nayak Y., Vijay Kumar D. (2012). Synthesis of 3-Methylflavones and Their Antioxidant and Antibacterial Activities. Med. Chem. Res..

[54] Zeinali M., Rezaee S.A., Hosseinzadeh H. (2017). An Overview on Immunoregulatory and Anti-Inflammatory Properties of Chrysin and Flavonoids Substances. Biomed. Pharmacother..

[55] Barreca M.M., Alessandro R., Corrado C. (2023). Effects of Flavonoids on Cancer, Cardiovascular and Neurodegenerative Diseases: Role of NF-ΚB Signaling Pathway. Int. J. Mol. Sci..

[56] Koosha S., Mohamed Z., Sinniah A., Alshawsh M.A. (2019). Investigation into the Molecular Mechanisms Underlying the Anti-Proliferative and Anti-Tumorigenesis Activities of Diosmetin against HCT-116 Human Colorectal Cancer. Sci. Rep..

[57] Cárdenas M., Marder M., Blank V.C., Roguin L.P. (2006). Antitumor Activity of Some Natural Flavonoids and Synthetic Derivatives on Various Human and Murine Cancer Cell Lines. Bioorg. Med. Chem..

[58] Daviaud C., Ferraz C.A.A., e Silva M.G., de Alencar Filho E.B., Evangelista Mendes de Lima S.Y., Dumontet V., Júnior L.J.Q., da Silva Almeida J.R.G., Picot L., Baranger K. (2025). Polymethoxyflavones from Gardenia Oudiepe and Semi-Synthetic Derivatives Reduce Nociception in Mice: Evidence for the Involvement of the MAPK Pathway. Biomed. Pharmacother..

[59] Mobbili G., Romaldi B., Sabbatini G., Amici A., Marcaccio M., Galeazzi R., Laudadio E., Armeni T., Minnelli C. (2023). Identification of Flavone Derivative Displaying a 4′-Aminophenoxy Moiety as Potential Selective Anticancer Agent in NSCLC Tumor Cells. Molecules.

[60] Khdera H.A., Saad S.Y., Moustapha A., Kandil F. (2022). Synthesis of New Flavonoid Derivatives Based on 3-Hydroxy-4′-Dimethylamino Flavone and Study the Activity of Some of Them as Antifungal. Heliyon.

[61] Kim H.M., Kwon M.H., Lee E.S., Ha K.B., Chung C.H. (2024). DA-6034 Ameliorates Hepatic Steatosis and Inflammation in High Fat Diet-Induced Obese Mice. J. Yeungnam Med. Sci..

