# Supplementary material for: Methyl Derivatives of Flavone as Potential Anti-Inflammatory Compounds
Source: Int J Mol Sci. 2025 Jan 16;26(2):729. doi: 10.3390/ijms26020729 (PMC11765865; doi:10.3390/ijms26020729)
Supplement: Supplementary file 1 [file ijms-26-00729-s001.zip › ijms-3404980-supplementary.pdf]

International Journal of *Molecular Sciences*

MDPI

Supplementary Materials

Methyl derivatives of flavone as potential anti-inflammatory compounds

Dagmara Jaworska [1,*], Małgorzata Kłósek [1], Joanna Bronikowska [1], Agnieszka Krawczyk-Łebek [2], Martyna Perz [2,3], Edyta Kostrzewa-Susłow [2] and Zenon P. Czuba [1]

[1] Department of Microbiology and Immunology; Faculty of Medical Sciences in Zabrze; Medical University of Silesia in Katowice, Jordana 19, 41-808 Zabrze, Poland; djaworska@sum.edu.pl (D.J); jbronikowska@sum.edu.pl (J.B.), mklosek@sum.edu.pl (M.K.); zczuba@sum.edu.pl (Z.P.C.)

[2] Department of Food Chemistry and Biocatalysis, Faculty of Biotechnology and Food Science, Wrocław University of Environmental and Life Sciences, 50-375 Wrocław, Poland; agnieszka.krawczyk-lebek@upwr.edu.pl (A.K.-Ł.); edyta.kostrzewa-suslow@upwr.edu.pl (E.K.-S.)

[3] Department of Biophysics and Neurobiology, Faculty of Medicine, Wrocław Medical University, Chałubińskiego 3A, 50-368 Wrocław, Poland; martyna.perz@umw.edu.pl (M.P.)

* Correspondence: djaworska@sum.edu.pl;

Content

Table S1. The viability of cell line RAW264.7 in the presence of tested compounds (2′-methylflavone (**5C**), 3′-methylflavone (**6C**), 4′-methylflavone (**7C**), 6-methylflavone (**8C**) and 6-methyl-8-nitroflavone (**12C**)); average, standard deviations, statistical significance. Statistical significance was analysed using HSD Tukey's test. Results marked in red are statistically significant.

Table S2. The production of nitric oxide by LPS-stimulated RAW 264.7 cells in the presence of flavone and its derivatives: 2′-methylflavone (**5C**), 3′-methylflavone (**6C**), 4′-methylflavone (**7C**), 6-methylflavone (**8C**) and 6-methyl-8-nitroflavone (**12C**); average, standard deviations, statistical significance. Statistical significance was analyzed using HSD Tukey's test. Results marked in red are statistically significant.

Table S3. The chemiluminescence of RAW 264.7 cells in the presence of flavone and its derivatives: 2′-methylflavone (**5C**), 3′-methylflavone (**6C**), 4′-methylflavone (**7C**), 6-methylflavone (**8C**) and 6-methyl-8-nitroflavone (**12C**); average, standard deviations, statistical significance. Statistical significance was analyzed using HSD Tukey's test. Results marked in red are statistically significant.

Table S4. The IL-1α, IL-1β, IL-6, IL-12p70 and TNF-α production by LPS stimulated RAW 264.7 cells in the presence of flavone and its derivatives: 2′-methylflavone (**5C**), 3′-methylflavone (**6C**), 4′-methylflavone (**7C**), 6-methylflavone (**8C**) and 6-methyl-8-nitroflavone (**12C**); average, standard deviations.

Table S5. The IL-1α production by LPS stimulated RAW 264.7 cells in the presence of flavone and its derivatives: 2′-methylflavone (**5C**), 3′-methylflavone (**6C**), 4′-methylflavone (**7C**), 6-methylflavone (**8C**) and 6-methyl-8-nitroflavone (**12C**); Statistical significance was analyzed using HSD Tukey's test. Results marked in red are statistically significant.

Table S6. The IL-1β production by LPS stimulated RAW 264.7 cells in the presence of flavone and its derivatives: 2′-methylflavone (**5C**), 3′-methylflavone (**6C**), 4′-methylflavone (**7C**), 6-methylflavone (**8C**) and 6-methyl-8-nitroflavone (**12C**); Statistical significance was analyzed using HSD Tukey's test. Results marked in red are statistically significant.

Table S7. The IL-6 production by LPS stimulated RAW 264.7 cells in the presence of flavone and its derivatives: 2′-methylflavone (**5C**), 3′-methylflavone (**6C**), 4′-methylflavone (**7C**), 6-methylflavone (**8C**) and 6-methyl-8-nitroflavone (**12C**); Statistical significance was analyzed using HSD Tukey's test. Results marked in red are statistically significant.

Table S8. The IL-12p70 production by LPS stimulated RAW 264.7 cells in the presence of flavone and its derivatives: 2′-methylflavone (**5C**), 3′-methylflavone (**6C**), 4′-methylflavone (**7C**), 6-methylflavone (**8C**) and 6-methyl-8-nitroflavone (**12C**); Statistical significance was analyzed using HSD Tukey's test. Results marked in red are statistically significant.

Table S9. The TNF-α production by LPS stimulated RAW 264.7 cells in the presence of flavone and its derivatives: 2′-methylflavone (**5C**), 3′-methylflavone (**6C**), 4′-methylflavone (**7C**), 6-methylflavone (**8C**) and 6-methyl-8-nitroflavone (**12C**); Statistical significance was analyzed using HSD Tukey's test. Results marked in red are statistically significant.

Table S1. The viability of cell line RAW 264.7 in the presence of tested compounds (flavone, 2′-methylflavone (**5C**), 3′-methylflavone (**6C**), 4′-methylflavone (**7C**), 6-methylflavone (**8C**) and 6-methyl-8-nitroflavone (**12C**)), average, standard deviations, statistical significance. Statistical significance was analyzed using HSD Tukey's test. Results marked in red are statistically significant.

	**RAW 264.7 cell viability (% of control)**			
**Flavone**	**LPS**		**LPS**	
**(µM)**	**0 ng/ml**	**SD**	**200 ng/ml**	**SD**
0	97.50	1.95	98.62	1.23
1	91.21	0.76	86.51	9.73
10	87.84	2.41	94.81	2.56
20	83.42	3.36	93.41	4.36

	**HSD Tukey's test; variable viability (%C). Probabilities for Post Hoc Tests. Error: Between MS = 17.907 df = 24.000**									
	**LPS**	**Flavone**	**{1}**	**{2}**	**{3}**	**{4}**	**{5}**	**{6}**	**{7}**	**{8}**
**No**	**(ng/ml)**	**(µM)**								
1	0	0		0.442997	0.060203	0.002028	0.999934	0.022534	0.983438	0.864221
2	0	1	0.442997		0.944362	0.202309	0.252108	0.761223	0.923734	0.994899
3	0	10	0.060203	0.944362		0.810792	0.026337	0.999797	0.319799	0.587745
4	0	20	0.002028	0.202309	0.810792		0.000872	0.964384	0.016556	0.047106
5	200	0	0.999934	0.252108	0.026337	0.000872		0.009415	0.899232	0.662047
6	200	1	0.022534	0.761223	0.999797	0.964384	0.009415		0.148943	0.329763
7	200	10	0.983438	0.923734	0.319799	0.016556	0.899232	0.148943		0.999728
8	200	20	0.864221	0.994899	0.587745	0.047106	0.662047	0.329763	0.999728	

	**RAW 264.7 cell viability (% of control)**			
	**LPS**		**LPS**	
**5C (µM)**	**0 ng/ml**	**SD**	**200 ng/ml**	**SD**
0	94.36	6.00	93.48	5.36
1	102.46	6.61	80.36	6.57
10	101.38	17.69	81.32	10.92
20	105.07	8.74	82.93	9.50

	**HSD Tukey's test; variable viability (%C). Probabilities for Post Hoc Tests. Error: Between MS = 93.804 df = 24.000**									
	**LPS**	**5C (µM)**	**{1}**	**{2}**	**{3}**	**{4}**	**{5}**	**{6}**	**{7}**	**{8}**
**No**	**(ng/ml)**									
1	0	0		0.929351	0.965916	0.765920	1.000000	0.475406	0.561184	0.705684
2	0	1	0.929351		1.000000	0.999929	0.885994	0.060065	0.080467	0.128536
3	0	10	0.965916	1.000000		0.999293	0.937643	0.083470	0.110612	0.172813
4	0	20	0.765920	0.999929	0.999293		0.691876	0.026018	0.035593	0.059268
5	200	0	1.000000	0.885994	0.937643	0.691876		0.553754	0.640914	0.778520
6	200	1	0.475406	0.060065	0.083470	0.026018	0.553754		1.000000	0.999936
7	200	10	0.561184	0.080467	0.110612	0.035593	0.640914	1.000000		0.999997
8	200	20	0.705684	0.128536	0.172813	0.059268	0.778520	0.999936	0.999997	

	**RAW 264.7 cell viability (% of control)**

**No**	**HSD Tukey's test; variable viability (%C). Probabilities for Post Hoc Tests. Error: Between MS = 93.072 df = 24.000**

	LPS		LPS	
**6C (µM)**	**0 ng/ml**	**SD**	**200 ng/ml**	**SD**
0	94.36	6.00	93.48	5.36
1	135.77	6.73	92.42	10.19
10	136.01	9.76	95.35	12.72
20	135.21	13.12	91.04	10.08

	LPS (ng/ml)	6C (µM)	{1}	{2}	{3}	{4}	{5}	{6}	{7}	{8}
1	0	0		0.000200	0.000195	0.000214	1.000000	0.999990	1.000000	0.999641
2	0	1	0.000200		1.000000	1.000000	0.000184	0.000171	0.000226	0.000161
3	0	10	0.000195	1.000000		1.000000	0.000181	0.000169	0.000219	0.000160
4	0	20	0.000214	1.000000	1.000000		0.000194	0.000178	0.000245	0.000165
5	200	0	1.000000	0.000184	0.000181	0.000194		1.000000	0.999992	0.999954
6	200	1	0.999990	0.000171	0.000169	0.000178	1.000000		0.999842	0.999999
7	200	10	1.000000	0.000226	0.000219	0.000245	0.999992	0.999842		0.998027
8	200	20	0.999641	0.000161	0.000160	0.000165	0.999954	0.999999	0.998027	

RAW 264.7 cell viability (% of control)

	LPS		LPS	
**7C (µM)**	**0 ng/ml**	**SD**	**200 ng/ml**	**SD**
0	98.18	2.06	98.98	1.10
1	87.67	4.10	96.08	7.66
10	94.46	5.19	94.66	2.91
20	89.73	4.86	93.67	2.65

HSD Tukey's test; variable viability (%C). Probabilities for Post Hoc Tests. Error: Between MS = 18.375 df = 24.000

No	LPS (ng/ml)	7C (µM)	{1}	{2}	{3}	{4}	{5}	{6}	{7}	{8}
1	0	0		0.035688	0.916119	0.144973	0.999994	0.996444	0.935268	0.805473
2	0	1	0.035688		0.363876	0.996899	0.019672	0.149101	0.330763	0.515888
3	0	10	0.916119	0.363876		0.766716	0.805078	0.999355	1.000000	0.999994
4	0	20	0.144973	0.996899	0.766716		0.086226	0.445761	0.730419	0.890527
5	200	0	0.999994	0.019672	0.805078	0.086226		0.976234	0.836619	0.654430
6	200	1	0.996444	0.149101	0.999355	0.445761	0.976234		0.999723	0.991795
7	200	10	0.935268	0.330763	1.000000	0.730419	0.836619	0.999723		0.999974
8	200	20	0.805473	0.515888	0.999994	0.890527	0.654430	0.991795	0.999974	

RAW 264.7 cell viability (% of control)

No **HSD Tukey's test; variable viability (%C). Probabilities for Post Hoc Tests. Error: Between MS = 7.3379 df = 24.000**

8C (μM)	LPS 0 ng/ml	SD	LPS 200 ng/ml	SD
0	96.67	3.82	96.94	3.71
1	91.03	3.03	91.43	1.83
10	89.13	2.51	95.08	2.36
20	91.88	1.71	95.71	1.75

	LPS (ng/ml)	8C (μM)	{1}	{2}	{3}	{4}	{5}	{6}	{7}	{8}
1	0	0		0.106319	0.012284	0.241932	1.000000	0.160080	0.989537	0.999564
2	0	1	0.106319		0.972077	0.999794	0.080430	0.999999	0.431532	0.264578
3	0	10	0.012284	0.972077		0.832697	0.008884	0.924056	0.077295	0.038341
4	0	20	0.241932	0.999794	0.832697		0.190400	0.999997	0.703997	0.501994
5	200	0	1.000000	0.080430	0.008884	0.190400		0.123156	0.974921	0.997872
6	200	1	0.160080	0.999999	0.924056	0.999997	0.123156		0.560160	0.367648
7	200	10	0.989537	0.431532	0.077295	0.703997	0.974921	0.560160		0.999974
8	200	20	0.999564	0.264578	0.038341	0.501994	0.997872	0.367648	0.999974	

	RAW 264.7 cell viability (% of control)			
12C (μM)	LPS 0 ng/ml	SD	LPS 200 ng/ml	SD
0	93.82	5.18	97.11	2.06
1	83.34	3.58	92.23	5.65
10	80.13	3.38	88.17	3.67
20	55.92	2.38	57.90	0.48

	HSD Tukey's test; variable viability (%C). Probabilities for Post Hoc Tests. Error: Between MS = 13.337 df = 24.000									
No	LPS (ng/ml)	12C (μM)	{1}	{2}	{3}	{4}	{5}	{6}	{7}	{8}
1	0	0		0.009256	0.000559	0.000147	0.899568	0.998382	0.392584	0.000147
2	0	1	0.009256		0.909643	0.000147	0.000530	0.037566	0.583599	0.000147
3	0	10	0.000559	0.909643		0.000147	0.000160	0.002102	0.076124	0.000147
4	0	20	0.000147	0.000147	0.000147		0.000147	0.000147	0.000147	0.993567
5	200	0	0.899568	0.000530	0.000160	0.000147		0.571959	0.036050	0.000147
6	200	1	0.998382	0.037566	0.002102	0.000147	0.571959		0.759526	0.000147
7	200	10	0.392584	0.583599	0.076124	0.000147	0.036050	0.759526		0.000147
8	200	20	0.000147	0.000147	0.000147	0.993567	0.000147	0.000147	0.000147	

Table S2. The production of nitric oxide by LPS-stimulated RAW 264.7 cells in the presence of flavone and its derivatives: 2′-methylflavone (**5C**), 3′-methylflavone (**6C**), 4′-methylflavone (**7C**), 6-methylflavone (**8C**) and 6-methyl-8-nitroflavone (**12C**); average, standard deviations, statistical significance. Statistical significance was analyzed using HSD Tukey's test. Results marked in red are statistically significant.

Nitric oxide (% of control)	Flavone	SD	5C	SD	6C	SD	7C	SD	8C	SD	12C	SD
**0 µM**	100.00	1.60	100.00	1.67	100.00	1.67	100.00	1.14	100.00	1.14	100.00	1.06
**1 µM**	92.78	5.72	66.57	3.85	72.80	5.32	93.28	5.21	82.06	4.26	92.10	4.34
**10 µM**	89.44	7.46	75.83	3.75	73.96	5.49	88.41	5.53	86.82	3.28	83.80	5.66
**20 µM**	80.31	5.85	75.27	5.08	68.73	5.22	81.03	6.45	83.23	1.78	79.08	2.04

HSD Tukey's test; variable NO (%C). Probabilities for Post Hoc Tests. Error: Between MS = 32.986 df = 54.000

No	Sample	{1}	{2}	{3}	{4}	{5}	{6}	{7}	{8}	{9}	{10}	{11}	{12}	{13}	{14}	{15}	{16}	{17}	{18}	{19}
1	Control		0.9493	0.4953	0.0016	0.0002	0.0002	0.0002	0.0002	0.0002	0.0002	0.9734	0.3315	0.0028	0.0061	0.1510	0.0147	0.8964	0.0221	0.0007
2	Flavone 1 µM	0.9493		1.0000	0.2200	0.0002	0.0129	0.0085	0.0040	0.0091	0.0003	1.0000	0.9998	0.3092	0.4685	0.9921	0.6695	1.0000	0.7601	0.1128
3	Flavone 10 µM	0.4953	1.0000		0.7376	0.0003	0.1184	0.0851	0.0384	0.0769	0.0024	1.0000	1.0000	0.8394	0.9391	1.0000	0.9879	1.0000	0.9957	0.5300
4	Flavone 20 µM	0.0016	0.2200	0.7376		0.1097	0.9998	0.9989	0.9637	0.9932	0.4677	0.1698	0.8760	1.0000	1.0000	0.9805	1.0000	0.3036	1.0000	1.0000
5	5C 1 µM	0.0002	0.0002	0.0003	0.1097		0.7169	0.8006	0.9945	0.9685	1.0000	0.0002	0.0004	0.0709	0.0361	0.0011	0.0159	0.0002	0.0105	0.2147
6	5C 10 µM	0.0002	0.0129	0.1184	0.9998	0.7169		1.0000	1.0000	1.0000	0.9785	0.0089	0.2077	0.9984	0.9874	0.4234	0.9375	0.0210	0.8896	1.0000
7	5C 20 µM	0.0002	0.0085	0.0851	0.9989	0.8006	1.0000		1.0000	1.0000	0.9906	0.0058	0.1548	0.9946	0.9708	0.3380	0.8905	0.0141	0.8252	1.0000
8	6C 1 µM	0.0002	0.0040	0.0384	0.9637	0.9945	1.0000	1.0000		1.0000	1.0000	0.0028	0.0715	0.9215	0.8188	0.1689	0.6514	0.0065	0.5612	0.9940
9	6C 10 µM	0.0002	0.0091	0.0769	0.9932	0.9685	1.0000	1.0000	1.0000		0.9997	0.0064	0.1354	0.9794	0.9306	0.2883	0.8172	0.0144	0.7413	0.9995
10	6C 20 µM	0.0002	0.0003	0.0024	0.4677	1.0000	0.9785	0.9906	1.0000	0.9997		0.0003	0.0050	0.3613	0.2340	0.0149	0.1317	0.0004	0.0971	0.6629
11	7C 1 µM	0.9734	1.0000	1.0000	0.1698	0.0002	0.0089	0.0058	0.0028	0.0064	0.0003		0.9993	0.2449	0.3874	0.9819	0.5838	1.0000	0.6803	0.0837
12	7C 10 µM	0.3315	0.9998	1.0000	0.8760	0.0004	0.2077	0.1548	0.0715	0.1354	0.0050	0.9993		0.9391	0.9848	1.0000	0.9985	1.0000	0.9997	0.7062
13	7C 20 µM	0.0028	0.3092	0.8394	1.0000	0.0709	0.9984	0.9946	0.9215	0.9794	0.3613	0.2449	0.9391		1.0000	0.9943	1.0000	0.4103	1.0000	1.0000
14	8C 1 µM	0.0061	0.4685	0.9391	1.0000	0.0361	0.9874	0.9708	0.8188	0.9306	0.2340	0.3874	0.9848	1.0000		0.9995	1.0000	0.5849	1.0000	1.0000
15	8C 10 µM	0.1510	0.9921	1.0000	0.9805	0.0011	0.4234	0.3380	0.1689	0.2883	0.0149	0.9819	1.0000	0.9943	0.9995		1.0000	0.9980	1.0000	0.9114
16	8C 20 µM	0.0147	0.6695	0.9879	1.0000	0.0159	0.9375	0.8905	0.6514	0.8172	0.1317	0.5838	0.9985	1.0000	1.0000	1.0000		0.7769	1.0000	0.9999
17	12C 1 µM	0.8964	1.0000	1.0000	0.3036	0.0002	0.0210	0.0141	0.0065	0.0144	0.0004	1.0000	1.0000	0.4103	0.5849	0.9980	0.7769		0.8523	0.1652
18	12C 10 µM	0.0221	0.7601	0.9957	1.0000	0.0105	0.8896	0.8252	0.5612	0.7413	0.0971	0.6803	0.9997	1.0000	1.0000	1.0000	1.0000	0.8523		0.9995
19	12C 20 µM	0.0007	0.1128	0.5300	1.0000	0.2147	1.0000	1.0000	0.9940	0.9995	0.6629	0.0837	0.7062	1.0000	1.0000	0.9114	0.9999	0.1652	0.9995	

Table S3. The chemiluminescence of RAW 264.7 cells in the presence of flavone and its derivatives: 2′-methylflavone (**5C**), 3′-methylflavone (**6C**), 4′-methylflavone (**7C**), 6-methylflavone (**8C**) and 6-methyl-8-nitroflavone (**12C**); average, standard deviations, statistical significance. Statistical significance was analyzed using HSD Tukey's test. Results marked in red are statistically significant.

**Chemiluminescence (% of control)**	**5C**	**SD**	**6C**	**SD**	**7C**	**SD**	**8C**	**SD**	**12C**	**SD**	**Flavone**	**SD**
**0 µM**	100.00	3.96	100.00	3.96	100.00	3.96	100.00	3.96	100.00	3.96	100.00	3.96
**1 µM**	100.61	8.75	93.88	7.66	110.98	4.94	122.24	10.37	104.80	7.97	106.32	6.46
**5 µM**	102.88	11.15	78.32	10.13	95.53	5.36	107.50	3.19	90.71	3.29	109.82	7.45
**10 µM**	70.82	2.09	38.54	1.97	59.13	4.66	63.49	3.40	85.06	3.33	56.80	3.37
**50 µM**	9.01	2.12	6.58	2.08	52.25	4.99	72.15	4.17	63.08	1.03	26.92	9.42

HSD Tukey's test; variable Chemiluminescence (%C). Probabilities for Post Hoc Tests. Error: Between MS = 48.558 df = 76.000

No	Sample	{1}	{2}	{3}	{4}	{5}	{6}	{7}	{8}	{9}	{10}	{11}	{12}	{13}	{14}	{15}	{16}	{17}	{18}	{19}	{20}	{21}	{22}	{23}	{24}	{25}
1	Control		0.999	0.878	0.000	0.000	1.000	1.000	0.000	0.000	0.999	0.041	0.000	0.000	0.731	1.000	0.000	0.000	0.001	0.992	0.000	0.000	1.000	0.924	0.172	0.000
2	Fla. 1 µM	0.999		1.000	0.000	0.000	1.000	1.000	0.000	0.000	0.673	0.002	0.000	0.000	1.000	0.875	0.000	0.000	0.210	1.000	0.000	0.000	1.000	0.241	0.010	0.000
3	Fla. 5 µM	0.878	1.000		0.000	0.000	0.972	0.999	0.000	0.000	0.209	0.000	0.000	0.000	1.000	0.401	0.000	0.000	0.676	1.000	0.000	0.000	1.000	0.039	0.001	0.000
4	Fla. 10 µM	0.000	0.000	0.000		0.000	0.000	0.000	0.439	0.000	0.000	0.000	0.064	0.000	0.000	0.000	1.000	1.000	0.000	0.000	0.999	0.412	0.000	0.000	0.000	0.999
5	Fla. 50 µM	0.000	0.000	0.000	0.000		0.000	0.000	0.000	0.045	0.000	0.000	0.700	0.009	0.000	0.000	0.000	0.001	0.000	0.000	0.000	0.000	0.000	0.000	0.000	0.000
6	5C 1 µM	1.000	1.000	0.972	0.000	0.000		1.000	0.000	0.000	0.999	0.076	0.000	0.000	0.910	1.000	0.000	0.000	0.007	0.999	0.000	0.000	1.000	0.941	0.247	0.000
7	5C 5 µM	1.000	1.000	0.999	0.000	0.000	1.000		0.000	0.000	0.978	0.019	0.000	0.000	0.994	0.998	0.000	0.000	0.034	1.000	0.000	0.000	1.000	0.712	0.082	0.000
8	5C 10 µM	0.000	0.000	0.000	0.439	0.000	0.000	0.000		0.000	0.003	0.757	0.000	0.000	0.000	0.001	0.775	0.114	0.000	0.000	0.998	1.000	0.000	0.024	0.407	0.996
9	5C 50 µM	0.000	0.000	0.000	0.000	0.045	0.000	0.000	0.000		0.000	0.000	0.000	1.000	0.000	0.000	0.000	0.000	0.000	0.000	0.000	0.000	0.000	0.000	0.000	0.000
10	6C 1 µM	0.999	0.673	0.209	0.000	0.000	0.999	0.978	0.003	0.000		0.829	0.000	0.000	0.120	1.000	0.000	0.000	0.000	0.497	0.000	0.021	0.862	1.000	0.982	0.000
11	6C 5 µM	0.041	0.002	0.000	0.000	0.000	0.076	0.019	0.757	0.000	0.829		0.000	0.000	0.000	0.608	0.002	0.000	0.000	0.000	0.038	0.951	0.005	0.994	1.000	0.030
12	6C 10 µM	0.000	0.000	0.000	0.064	0.700	0.000	0.000	0.000	0.000	0.000	0.000		0.000	0.000	0.000	0.015	0.636	0.000	0.000	0.000	0.000	0.000	0.000	0.000	0.001
13	6C 50 µM	0.000	0.000	0.000	0.000	0.009	0.000	0.000	0.000	1.000	0.000	0.000	0.000		0.000	0.000	0.000	0.000	0.000	0.000	0.000	0.000	0.000	0.000	0.000	0.000
14	7C 1 µM	0.731	1.000	1.000	0.000	0.000	0.910	0.994	0.000	0.000	0.120	0.000	0.000	0.000		0.258	0.000	0.000	0.827	1.000	0.000	0.000	0.999	0.019	0.000	0.000
15	7C 5 µM	1.000	0.875	0.401	0.000	0.000	1.000	0.998	0.001	0.000	1.000	0.608	0.000	0.000	0.258		0.000	0.000	0.000	0.738	0.000	0.007	0.969	1.000	0.903	0.000
16	7C 10 µM	0.000	0.000	0.000	1.000	0.000	0.000	0.000	0.775	0.000	0.000	0.002	0.015	0.000	0.000	0.000		0.999	0.000	0.000	1.000	0.727	0.000	0.000	0.000	1.000
17	7C 50 µM	0.000	0.000	0.000	1.000	0.001	0.000	0.000	0.114	0.000	0.000	0.000	0.636	0.000	0.000	0.000	0.999		0.000	0.000	0.907	0.112	0.000	0.000	0.000	0.933
18	8C 1 µM	0.001	0.210	0.676	0.000	0.000	0.007	0.034	0.000	0.000	0.000	0.000	0.000	0.000	0.827	0.000	0.000	0.000		0.342	0.000	0.000	0.101	0.000	0.000	0.000
19	8C 5 µM	0.992	1.000	1.000	0.000	0.000	0.999	1.000	0.000	0.000	0.497	0.000	0.000	0.000	1.000	0.738	0.000	0.000	0.342		0.000	0.000	1.000	0.140	0.004	0.000

20	8C 10 µM	0.000	0.000	0.000	0.999	0.000	0.000	0.000	0.998	0.000	0.000	0.038	0.000	0.000	0.000	0.000	1.000	0.907	0.000	0.000		0.994	0.000	0.000	0.008	1.000
21	8C 50 µM	0.000	0.000	0.000	0.412	0.000	0.000	0.000	1.000	0.000	0.021	0.951	0.000	0.000	0.000	0.007	0.727	0.112	0.000	0.000	0.994		0.000	0.115	0.741	0.990
22	12C 1 µM	1.000	1.000	1.000	0.000	0.000	1.000	1.000	0.000	0.000	0.862	0.005	0.000	0.000	0.999	0.969	0.000	0.000	0.101	1.000	0.000	0.000		0.429	0.027	0.000
23	12C 5 µM	0.924	0.241	0.039	0.000	0.000	0.941	0.712	0.024	0.000	1.000	0.994	0.000	0.000	0.019	1.000	0.000	0.000	0.000	0.140	0.000	0.115	0.429		1.000	0.000
24	12C 10 µM	0.172	0.010	0.001	0.000	0.000	0.247	0.082	0.407	0.000	0.982	1.000	0.000	0.000	0.000	0.903	0.000	0.000	0.000	0.004	0.008	0.741	0.027	1.000		0.006
25	12C 50 µM	0.000	0.000	0.000	0.999	0.000	0.000	0.000	0.996	0.000	0.000	0.030	0.001	0.000	0.000	0.000	1.000	0.933	0.000	0.000	1.000	0.990	0.000	0.000	0.006	

Table S4. The IL-1α, IL-1β, IL-6, IL-12p70 and TNF-α production by LPS stimulated RAW 264.7 cells in the presence of flavone and its derivatives: 2′-methylflavone (**5C**), 3′-methylflavone (**6C**), 4′-methylflavone (**7C**), 6-methylflavone (**8C**) and 6-methyl-8-nitroflavone (**12C**); average, standard deviations.

**Cytokine production (% of control)**	**IL-1α**	**SD**	**IL-1β**	**SD**	**IL-6**	**SD**	**IL-12p70**	**SD**	**TNF-α**	**SD**
Control	29.01	10.37	34.68	5.4061	0.9548	0.5879	25.681	12.774	2.087	0.9003
Control DMSO	100.00	8.49	100	8.2663	100	9.3616	100	12.033	100	8.4095
5C 1 µM	60.81	7.72	78.162	6.5176	64.608	9.8115	65.858	10.914	34.439	5.4851
5C 20 µM	65.80	12.66	61.591	4.5158	47.781	3.0335	69.125	6.7773	26.755	4.4277
6C 1 µM	58.84	7.11	98.8	1.3158	99.43	7.4656	86.32	5.7458	62.612	4.3385
6C 20 µM	55.11	2.87	76.498	4.8531	75.744	1.6922	81.685	1.9973	44.02	3.4706
7C 1 µM	74.05	3.81	102.97	13.302	109.7	14.041	95.049	4.2341	80.26	10.921
7C 20 µM	77.57	21.02	95.056	4.6385	77.071	1.3873	87.135	5.4692	70.131	4.0819
8C 1 µM	76.62	16.54	100.35	9.2261	92.176	3.8687	85.933	9.361	92.813	5.1282
8C 20 µM	67.32	21.75	94.968	12.33	85.193	13.966	79.252	4.614	85.465	4.1363
12C 1 µM	48.71	10.84	91.55	7.9501	26.034	1.2462	75.24	3.3888	56.732	4.678
12C 20 µM	55.94	6.03	81.924	1.3994	27.524	0.9162	73.567	10.681	56.633	4.9488
Flavone 1 µM	116.10	16.04	104.48	9.2144	130.25	21.677	88.274	9.1854	59.355	3.9758
Flavone 20 µM	126.18	26.56	100.78	2.1937	82.782	6.8007	95.512	7.0731	44.16	3.5212

Table S5. The IL-1α production by LPS stimulated RAW 264.7 cells in the presence of flavone and its derivatives: 2′-methylflavone (**5C**), 3′-methylflavone (**6C**), 4′-methylflavone (**7C**), 6-methylflavone (**8C**) and 6-methyl-8-nitroflavone (**12C**); Statistical significance, HSD Tukey's test. Results marked in red are statistically significant.

HSD Tukey's test; variable IL-1α (%C). Probabilities for Post Hoc Tests. Error: Between MS = 180.64 df = 32.000

No	Sample	{1}	{2}	{3}	{4}	{5}	{6}	{7}	{8}	{9}	{10}	{11}	{12}	{13}
1	Control		0.837419	0.200655	0.006700	0.027836	0.003742	0.001250	0.210636	0.405922	0.345488	0.041882	0.000269	0.001598
2	Flavone 1 μM	0.837419		0.999119	0.001165	0.003857	0.000746	0.000356	0.026849	0.057832	0.047244	0.005542	0.000174	0.000412
3	Flavone 20 μM	0.200655	0.999119		0.000202	0.000396	0.000175	0.000151	0.002478	0.005802	0.004601	0.000529	0.000141	0.000154
4	5C 1 μM	0.006700	0.001165	0.000202		1.000000	1.000000	0.999998	0.989520	0.938560	0.958880	0.999991	0.995174	1.000000
5	5C 20 μM	0.027836	0.003857	0.000396	1.000000		0.999980	0.998447	0.999882	0.996230	0.998265	1.000000	0.930170	0.999286
6	6C 1 μM	0.003742	0.000746	0.000175	1.000000	0.999980		1.000000	0.968938	0.877451	0.910266	0.999844	0.999079	1.000000
7	6C 20 μM	0.001250	0.000356	0.000151	0.999998	0.998447	1.000000		0.869338	0.696264	0.748861	0.994779	0.999992	1.000000
8	7C 1 μM	0.210636	0.026849	0.002478	0.989520	0.999882	0.968938	0.869338		1.000000	1.000000	0.999986	0.526685	0.899514
9	7C 20 μM	0.405922	0.057832	0.005802	0.938560	0.996230	0.877451	0.696264	1.000000		1.000000	0.998961	0.333869	0.742359
10	8C 1 μM	0.345488	0.047244	0.004601	0.958880	0.998265	0.910266	0.748861	1.000000	1.000000		0.999598	0.381963	0.791720
11	8C 20 μM	0.041882	0.005542	0.000529	0.999991	1.000000	0.999844	0.994779	0.999986	0.998961	0.999598		0.882212	0.997228
12	12C 1 μM	0.000269	0.000174	0.000141	0.995174	0.930170	0.999079	0.999992	0.526685	0.333869	0.381963	0.882212		0.999971
13	12C 20 μM	0.001598	0.000412	0.000154	1.000000	0.999286	1.000000	1.000000	0.899514	0.742359	0.791720	0.997228	0.999971	

Table S6. The IL-1β production by LPS stimulated RAW 264.7 cells in the presence of flavone and its derivatives: 2′-methylflavone (**5C**), 3′-methylflavone (**6C**), 4′-methylflavone (**7C**), 6-methylflavone (**8C**) and 6-methyl-8-nitroflavone (**12C**); Statistical significance, HSD Tukey's test. Results marked in red are statistically significant.

HSD Tukey's test; variable IL-1β (%C). Probabilities for Post Hoc Tests. Error: Between MS = 69.849 df = 32.000

No	**Sample**	**{1}**	**{2}**	**{3}**	**{4}**	**{5}**	**{6}**	**{7}**	**{8}**	**{9}**	**{10}**	**{11}**	**{12}**	**{13}**
1	Control		0.999926	0.999780	0.003016	0.000144	0.993418	0.001438	1.000000	0.840376	0.832993	1.000000	0.472599	0.015973
2	Flavone 1 µM	0.999926		1.000000	0.079917	0.000268	1.000000	0.046081	1.000000	0.999203	0.999086	1.000000	0.962440	0.240137
3	Flavone 20 µM	0.999780	1.000000		0.092546	0.000296	1.000000	0.053865	1.000000	0.999628	0.999567	1.000000	0.973748	0.270227
4	5C 1 µM	0.003016	0.079917	0.092546		0.452685	0.167597	1.000000	0.045023	0.423860	0.431635	0.048045	0.747621	0.999996
5	5C 20 µM	0.000144	0.000268	0.000296	0.452685		0.000503	0.608626	0.000201	0.001872	0.001934	0.000206	0.007025	0.182654
6	6C 1 µM	0.993418	1.000000	1.000000	0.167597	0.000503		0.102124	0.999986	0.999996	0.999995	0.999992	0.996443	0.425459
7	6C 20 µM	0.001438	0.046081	0.053865	1.000000	0.608626	0.102124		0.025223	0.290873	0.297199	0.026993	0.594897	0.999783
8	7C 1 µM	1.000000	1.000000	1.000000	0.045023	0.000201	0.999986	0.025223		0.992321	0.991564	1.000000	0.889960	0.148862
9	7C 20 µM	0.840376	0.999203	0.999628	0.423860	0.001872	0.999996	0.290873	0.992321		1.000000	0.993767	0.999998	0.769206
10	8C 1 µM	0.832993	0.999086	0.999567	0.431635	0.001934	0.999995	0.297199	0.991564	1.000000		0.993130	0.999998	0.776475
11	8C 20 µM	1.000000	1.000000	1.000000	0.048045	0.000206	0.999992	0.026993	1.000000	0.993767	0.993130		0.900503	0.157337
12	12C 1 µM	0.472599	0.962440	0.973748	0.747621	0.007025	0.996443	0.594897	0.889960	0.999998	0.999998	0.900503		0.964208
13	12C 20 µM	0.015973	0.240137	0.270227	0.999996	0.182654	0.425459	0.999783	0.148862	0.769206	0.776475	0.157337	0.964208	

Table S7. The IL-6 production by LPS stimulated RAW 264.7 cells in the presence of flavone and its derivatives: 2′-methylflavone (**5C**), 3′-methylflavone (**6C**), 4′-methylflavone (**7C**), 6-methylflavone (**8C**) and 6-methyl-8-nitroflavone (**12C**); Statistical significance, HSD Tukey's test. Results marked in red are statistically significant.

HSD Tukey's test; variable IL-6 (%C). Probabilities for Post Hoc Tests. Error: Between MS = 90.141 df = 32.000

No	Sample	{1}	{2}	{3}	{4}	{5}	{6}	{7}	{8}	{9}	{10}	{11}	{12}	{13}
1	Control		0.002296	0.287850	0.000335	0.000140	1.000000	0.026774	0.937423	0.044464	0.987249	0.507489	0.000140	0.000140
2	Flavone 1 µM	0.002296		0.000176	0.000140	0.000140	0.018730	0.000141	0.322855	0.000143	0.001623	0.000238	0.000140	0.000140
3	Flavone 20 µM	0.287850	0.000176		0.504250	0.004609	0.632302	0.999216	0.063140	0.999905	0.989171	1.000000	0.000140	0.000141
4	5C 1 µM	0.000335	0.000140	0.504250		0.617276	0.004898	0.959836	0.000237	0.914633	0.051983	0.320693	0.001364	0.002269
5	5C 20 µM	0.000140	0.000140	0.004609	0.617276		0.000146	0.046108	0.000140	0.030553	0.000268	0.002029	0.248361	0.343201
6	6C 1 µM	1.000000	0.018730	0.632302	0.004898	0.000146		0.154676	0.977991	0.215181	0.998950	0.817379	0.000140	0.000140
7	6C 20 µM	0.026774	0.000141	0.999216	0.959836	0.046108	0.154676		0.006597	1.000000	0.650375	0.988629	0.000154	0.000166
8	7C 1 µM	0.937423	0.322855	0.063140	0.000237	0.000140	0.977991	0.006597		0.010328	0.558748	0.124741	0.000140	0.000140
9	7C 20 µM	0.044464	0.000143	0.999905	0.914633	0.030553	0.215181	1.000000	0.010328		0.756045	0.996955	0.000148	0.000155
10	8C 1 µM	0.987249	0.001623	0.989171	0.051983	0.000268	0.998950	0.650375	0.558748	0.756045		0.999274	0.000140	0.000140
11	8C 20 µM	0.507489	0.000238	1.000000	0.320693	0.002029	0.817379	0.988629	0.124741	0.996955	0.999274		0.000140	0.000140
12	12C 1 µM	0.000140	0.000140	0.000140	0.001364	0.248361	0.000140	0.000154	0.000140	0.000148	0.000140	0.000140		1.000000
13	12C 20 µM	0.000140	0.000140	0.000141	0.002269	0.343201	0.000140	0.000166	0.000140	0.000155	0.000140	0.000140	1.000000	

Table S8. The IL-12p70 production by LPS stimulated RAW 264.7 cells in the presence of flavone and its derivatives: 2′-methylflavone (**5C**), 3′-methylflavone (**6C**), 4′-methylflavone (**7C**), 6-methylflavone (**8C**) and 6-methyl-8-nitroflavone (**12C**); Statistical significance, HSD Tukey's test. Results marked in red are statistically significant.

HSD Tukey's test; variable IL-12p70 (%C). Probabilities for Post Hoc Tests. Error: Between MS = 73.757 df = 32.000

No	Sample	{1}	{2}	{3}	{4}	{5}	{6}	{7}	{8}	{9}	{10}	{11}	{12}	{13}
1	Control		0.695850	0.999819	0.000200	0.000497	0.475681	0.115235	0.999513	0.567421	0.011840	0.044329	0.007622	0.003522
2	Flavone 1 µM	0.695850		0.997340	0.115740	0.282771	1.000000	0.998909	0.998580	1.000000	0.871724	0.982439	0.805806	0.664695
3	Flavone 20 µM	0.999819	0.997340		0.009797	0.031723	0.979681	0.742307	1.000000	0.990371	0.272115	0.520675	0.212498	0.133109
4	5C 1 µM	0.000200	0.115740	0.009797		0.999999	0.202016	0.560802	0.011625	0.161435	0.951443	0.777902	0.976207	0.995296
5	5C 20 µM	0.000497	0.282771	0.031723	0.999999		0.436426	0.839903	0.037231	0.368006	0.997825	0.958281	0.999472	0.999981
6	6C 1 µM	0.475681	1.000000	0.979681	0.202016	0.436426		0.999970	0.986519	1.000000	0.958658	0.997861	0.923449	0.826396
7	6C 20 µM	0.115235	0.998909	0.742307	0.560802	0.839903	0.999970		0.780370	0.999834	0.999825	1.000000	0.999116	0.992625
8	7C 1 µM	0.999513	0.998580	1.000000	0.011625	0.037231	0.986519	0.780370		0.994075	0.304469	0.563685	0.239783	0.152243
9	7C 20 µM	0.567421	1.000000	0.990371	0.161435	0.368006	1.000000	0.999834	0.994075		0.930003	0.994274	0.881913	0.763876
10	8C 1 µM	0.011840	0.871724	0.272115	0.951443	0.997825	0.958658	0.999825	0.304469	0.930003		1.000000	1.000000	1.000000
11	8C 20 µM	0.044329	0.982439	0.520675	0.777902	0.958281	0.997861	1.000000	0.563685	0.994274	1.000000		0.999994	0.999745
12	12C 1 µM	0.007622	0.805806	0.212498	0.976207	0.999472	0.923449	0.999116	0.239783	0.881913	1.000000	0.999994		1.000000
13	12C 20 µM	0.003522	0.664695	0.133109	0.995296	0.999981	0.826396	0.992625	0.152243	0.763876	1.000000	0.999745	1.000000	

Table S9. The TNF-α production by LPS stimulated RAW 264.7 cells in the presence of flavone and its derivatives: 2′-methylflavone (**5C**), 3′-methylflavone (**6C**), 4′-methylflavone (**7C**), 6-methylflavone (**8C**) and 6-methyl-8-nitroflavone (**12C**); Statistical significance, HSD Tukey's test. Results marked in red are statistically significant.

HSD Tukey's test; variable TNFα (%C). Probabilities for Post Hoc Tests. Error: Between MS = 38.585. df = 32.000

No	Sample	{1}	{2}	{3}	{4}	{5}	{6}	{7}	{8}	{9}	{10}	{11}	{12}	{13}
1	Control		0.000140	0.000140	0.000140	0.000140	0.000140	0.000140	0.002371	0.000141	0.865394	0.057882	0.000140	0.000140
2	Flavone 1 µM	0.000140		0.368495	0.004998	0.000194	0.996292	0.353069	0.004202	0.376572	0.000141	0.000344	1.000000	1.000000
3	Flavone 20 µM	0.000140	0.368495		0.774154	0.069224	0.042861	1.000000	0.000141	0.000944	0.000140	0.000140	0.420364	0.432148
4	5C 1 µM	0.000140	0.004998	0.774154		0.941926	0.000358	0.789509	0.000140	0.000141	0.000140	0.000140	0.006336	0.006666
5	5C 20 µM	0.000140	0.000194	0.069224	0.941926		0.000141	0.073673	0.000140	0.000140	0.000140	0.000140	0.000212	0.000216
6	6C 1 µM	0.000140	0.996292	0.042861	0.000358	0.000141		0.040128	0.062050	0.949877	0.000202	0.004719	0.992536	0.991398
7	6C 20 µM	0.000140	0.353069	1.000000	0.789509	0.073673	0.040128		0.000141	0.000882	0.000140	0.000140	0.403863	0.415446
8	7C 1 µM	0.002371	0.004202	0.000141	0.000140	0.000140	0.062050	0.000141		0.727236	0.422513	0.997475	0.003317	0.003152
9	7C 20 µM	0.000141	0.376572	0.000944	0.000141	0.000140	0.949877	0.000882	0.727236		0.005161	0.164937	0.327726	0.317545
10	8C 1 µM	0.865394	0.000141	0.000140	0.000140	0.000140	0.000202	0.000140	0.422513	0.005161		0.957353	0.000141	0.000141
11	8C 20 µM	0.057882	0.000344	0.000140	0.000140	0.000140	0.004719	0.000140	0.997475	0.164937	0.957353		0.000295	0.000286
12	12C 1 µM	0.000140	1.000000	0.420364	0.006336	0.000212	0.992536	0.403863	0.003317	0.327726	0.000141	0.000295		1.000000
13	12C 20 µM	0.000140	1.000000	0.432148	0.006666	0.000216	0.991398	0.415446	0.003152	0.317545	0.000141	0.000286	1.000000	
